# QTL Mapping of Flowering and Fruiting Traits in Olive

**DOI:** 10.1371/journal.pone.0062831

**Published:** 2013-05-17

**Authors:** Inès Ben Sadok, Jean-Marc Celton, Laila Essalouh, Amal Zine El Aabidine, Gilbert Garcia, Sebastien Martinez, Naziha Grati-Kamoun, Ahmed Rebai, Evelyne Costes, Bouchaib Khadari

**Affiliations:** 1 INRA, UMR 1334 Amélioration Génétique et Adaptation des Plantes méditerranéennes et tropicales (AGAP), Campus Cirad- TA-A-108/03, Montpellier, France; 2 Montpellier SupAgro, UMR AGAP, Campus Cirad- TA-A-108/03, Montpellier, France; 3 Institut de l’olivier de Sfax, Sfax, Tunisie; 4 Université des sciences de Sfax, Sfax, Tunisie; 5 INRA, UMR1345 Institut de Recherche en Horticulture et Semences, SFR 4207 QUASAV, PRES L’UNAM, Angers, France; 6 AgroCampus-Ouest, UMR1345 Institut de Recherche en Horticulture et Semences, Angers, France; 7 Université d’Angers, UMR1345 Institut de Recherche en Horticulture and Semences, Beaucouzé, France; University of New England, Australia

## Abstract

One of the challenge fruit growers are facing is to balance between tree production and vegetative growth from year to year. To investigate the existence of genetic determinism for reproductive behaviour in olive tree, we studied an olive segregating population derived from a cross between ‘Olivière’ and ‘Arbequina’ cultivars. Our strategy was based on (i) an annual assessment of individual trees yield, and (ii) a decomposition of adult growth units at the crown periphery into quantitative variables related to both flowering and fruiting process in relation to their growth and branching. Genetic models, including the year, genotype effects and their interactions, were built with variance function and correlation structure of residuals when necessary. Among the progeny, trees were either ‘ON’ or ‘OFF’ for a given year and patterns of regular vs. irregular bearing were revealed. Genotype effect was significant on yield but not for flowering traits at growth unit (GU) scale, whereas the interaction between genotype and year was significant for both traits. A strong genetic effect was found for all fruiting traits without interaction with the year. Based on the new constructed genetic map, QTLs with small effects were detected, revealing multigenic control of the studied traits. Many were associated to alleles from ‘Arbequina’. Genetic correlations were found between Yield and Fruit set at GU scale suggesting a common genetic control, even though QTL co-localisations were in spe`cific years only. Most QTL were associated to flowering traits in specific years, even though reproductive traits at GU scale did not capture the bearing status of the trees in a given year. Results were also interpreted with respect to ontogenetic changes of growth and branching, and an alternative sampling strategy was proposed for capturing tree fruiting behaviour. Regular bearing progenies were identified and could constitute innovative material for selection programs.

## Introduction

Once they reach adulthood, perennial species undergo several morphological and physiological changes from vegetative to reproductive phases. The process of flowering involves converting into flowers or inflorescences a portion or sometimes all meristems that would otherwise produce vegetative shoots [1]. However, a minimum of vegetative development is needed to reach the adult phase and the ability to develop flowers [2], [3]. The transition from a vegetative meristem to a reproductive one is usually divided into two phases: i) the induction phase, that involves biochemical modifications leading the apical bud to the formation of a reproductive structure, and ii) the differentiation phase during which the tissues of flower organs are formed [4]. Flower initiation is usually limited by concomitant heavy cropping which can lead to alternate bearing [5].

Alternate bearing, described as a sequence of heavy yields followed by light ones over several years, affects several temperate, tropical and sub-tropical fruit tree species such as apple, pear, apricot, coffee, and mangos [5]. The inhibition of floral induction by developing fruits has been pointed as playing a central role in alternate or irregular bearing but the physiological causes of alternation are still under investigation [5], [6]. The hormonal control of floral induction has been reported in perennial fruit species. Gibberellins (GA) and auxins could potentially act as inhibiting signals, whereas cytokinins are likely to enhance floral induction [7]. Using apple as a model, Guitton et al. [8] demonstrated the genetic determinism of fruiting behaviour and identified several genes related to metabolism, degradation and transport of GA and auxins, that co-localized with QTLs for biennial bearing. These authors have showed that the control of fruit bearing is a complex multigenic process, influenced by both tree age and climatic year. In addition, the development of new shoots plays a central role in the complex interactions determining vegetative and reproductive growth in woody plants. In fact, in the fruiting year, resources are principally oriented to flowering and fruiting at the expense of vegetative growth [5], [9] leading to a reduced vegetative growth and possibly a reduced floral induction in the following year.

Quantifying the regularity of fruit tree production usually relies on evaluation over consecutive years and can be summarized through indices such as the biennial bearing index (BBI). BBI initially proposed by Hoblyn et al. [10] and further renamed by Wilcox [11] has been used in several species such as apple [8], citrus [12] and pistachio [13]. Its calculation accounts for the intensity of deviation of tree yield, over successive years whatever its sign.

In the olive tree (*Olea europaea L*. *subsp. europaea*), cyclical changes in crop production are particularly severe and cause drastic economical issues [14]. Similar hypothesis about the inhibition of the floral induction by developing fruits as well as the role of vegetative growth under bearing condition are privileged [15], [16]. Fruit production has a competitive effect on new vegetative development, and thus on the formation of potential flowering sites as olive inflorescences are usually developed from the buds formed in the leaf axils of current growing season’s shoots [17]–[19]. Fruits representing more than 65% of the seasonal dry matter accumulated in the fruiting shoots, reduce shoot growth in bearing years and affects fruit ripening [20]–[21]. Both reproductive shoot percentage and flowering have been reported to be substantially higher in previously non-bearing olive trees [18]. The relationship between the reproductive behaviour and new shoot development has been studied on the olive cultivar ‘Hojiblanca’, showing that even though new shoots development was higher for non-bearing years, shoots were predominantly of short length during both bearing and non-bearing years [22].

Besides biennial bearing habit, olive fruit production is characterized by a massive abscission of young fruits in post-bloom period. The capacity to set fruit results from an equilibrium between flowers within the inflorescence and is modulated by fertilization of ovaries and by competition among fruitlets [15], [23]. Moreover, fruit set is not only controlled by competition within each inflorescence but is dependent on the global fruiting potential of the tree [24]. Another potential factor affecting olive tree cropping is the abundance of staminate flowers relative to hermaphroditic ones, and/or their irregular distribution in the canopy [25], [26]. Staminate flowers are incompetent to set fruits due to their inability to complete pistil development. Moreover, environmental and nutritional stress between bud burst and 6 weeks before anthesis reduce the number of flowers per inflorescences and increases pistil abortion [26], [27].

To date, most reproductive behaviour studies have focused on mature olive cultivars, giving insights on the physiological processes and environmental constrains affecting flowering and fruiting habits. Very little is known about the segregation of reproductive development traits and the morphogenetic factors impacting fruiting behaviour in the olive tree. Knowledge on olive genetic remains scarce, due to the long generation time (juvenility phase can last up to 15 years). This is also owed to the olive high heterozygosity and chromosome number (2 n = 46) for a genome size of about 3,120 Mbp [28].

In the present study, we investigated the genetic basis of the reproductive development in a F1 olive tree progeny derived from a cross between ‘Olivière’ and ‘Arbequina’ cultivars, with contrasted architectural traits and bearing behaviour. Our strategy was based on the quantification of the regularity of fruit tree production among progenies and the monitoring of the flowering and fruiting habit changes on adult growth units selected at the crown periphery with respect to trees ontogeny. We investigated major factors impacting reproductive traits and the relations between tree bearing status i.e. ‘ON’ or ‘OFF’ and the number of inflorescences and fruits developed on growth units. After building a new genetic map, QTLs mapping was finally carried out on the heritable traits.

## Materials and Methods

### Plant Material

The studied progeny derived from ‘Olivière’×‘Arbequina’ cross and counts 160 genotypes, each replicated twice [29]. Parental cultivars were chosen for their contrasting architecture as well as their different flowering and fruiting potential. ‘Olivière’ is a vigorous male sterile French cultivar with a fast growth rate and a rapid entrance in production [30]. ‘Arbequina’ is a very productive Spanish cultivar adapted for high density [31]. In 2005, progenies and parents were planted at INRA-Montpellier experimental station using a random experimental design. After plantation, trunks were cut back to 50 cm to homogenize the plantation and trees were not pruned afterwards. Trees were regularly irrigated and phytosanitary condition was controlled by conventional practices all over the experiment.

### Phenotyping

In 2008, 74% of progenies have started to produce inflorescences and fruits. Once all trees had entered into reproductive period in 2009, we initiated the study of their flowering and fruiting behavior. For studying ‘Olivière’×‘Arbequina’ progenies bearing behavior at the whole tree scale, fruits were harvested and weighed for each individual. Four harvesting campaigns were performed from 2008 and until 2011, i.e. from the forth to the seventh year of growth. Since, fruit ripening was relatively late in time among the progeny, the harvesting period was in December of each year. The mass of 100 fruits was determined for each tree in order to estimate the mass of individual fruits. This information was also used to estimate fruits dropped on the ground. The total fruit weight per tree (Yield) was calculated as the sum of the weight of harvested fruits on the tree and the weight of the fruits on the ground.

To quantify growth and branching as well as flowering at the growth unit (GU) scale, two one-year-old GUs (long or medium) were selected per tree at the tree periphery [29]. Each GU was described twice per year, at flowering and fruit set periods during 3 consecutive years (2009–2011), (Figure S1). The number of inflorescences and fruits born at the leaf axils along these GUs or along their laterals were noted Inflo (or Fruit)_direct and Inflo (or Fruit)_AS, respectively. We considered the capacity for an inflorescence to develop at least one fruit as a measure of fruit set. The percentage of fruit set was calculated afterwards as the ratio between the number of inflorescences with at least one fruit and the total number of inflorescences along the GUs (Total_Fruitset), or the direct inflorescences (Fruitset_direct), or inflorescences along GUs sylleptic laterals (Fruitset_AS). All measured and calculated descriptors of bearing behaviour were classified according to the observation scale: whole tree and growth unit (Table 1).

**Table 1:** Variables related to olive flowering and fruiting habit collected at tree and GUs scale.

Observation scale	Measured and calculated Variables	Abbreviations	Formula
Whole tree	Mass of fruits at harvest	Yield	
	Nb of inflorescences (or Fruit)	Inflo(or Fruit) _tot	Inflo(or Fruit) _direct+Inflo(or Fruit) _AS
	Nb of direct inflorescences(or Fruit)	Inflo(or Fruit) _direct	
**One year- old floral** **Growth Units**	Nb of inflorescences(or Fruit) on axillary.shoots	Inflo(or Fruit) _AS	
	id. on long, medium, short axillary. shoots	Inflo_L; Inflo_M; Inflo_S	
	Percentage of fruiting	Total_Fruitset	Fruit_tot/Inflo_tot
	Percentage of direct fruit set	Fruitset_direct	Fruit_direct/Inflo_direct
	Percentage of laterals fruit set	Fruitset_AS	Fruit_AS/Inflo_AS

### Qualitative Classification of Progenies Bearing Behaviour and BBI Calculation

Considering the mean Yield value per genotype, the sequence of the four years of production (2008–2011), was graphically drawn. Only genotypes having started producing in 2008 were considered i.e. a total of 141 progenies. Genotypes were then classified according to their yield evolution from a year *n* to *n*+1, as proposed by Guitton et al. [8]. For each pair of years, the direction of yield variation was coded by ‘+’ or ‘−’ symbols. Genotypes following the same pattern were clustered manually, and the mean Yield value of all genotypes per year was calculated for each cluster. Genotypes exhibiting lower or higher production in years *n*-1 and *n*+1 than in year n were considered as irregular. When production was increasing stably over years, genotypes were qualified as regular bearing.

Alternate bearing behavior of each genotype was quantified by the BBI calculation according to the formula below [10], [11]:
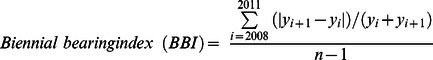
Where *y_i_* refers to the yield for year *i*, and *n* the number of studied years of production (in our case, *n* = 4).

### Statistical Analysis

At tree scale, yields were analyzed on 282 trees (141 progenies repeated twice), previously used for manual classification. At GU scale, the analysis of flowering and fruiting variables was restricted to 240 trees (120 progenies repeated twice), on which data were available over the 3 years (2009 to 2011). Even though GUs phenotyped from a year to another were different, the studied variables may be correlated as the same trees were considered for the three consecutive years. Thus, variances homogeneity and covariance between successive years were examined by Levene’s test [32]. Because distribution of flowering and fruiting variables was not Gaussian, all the variables were transformed with a square root transformation.

At both the GU scale and whole tree scale, the effect of genotype and year factors and their interactions on the reproductive behaviour variables were estimated according to the following mixed model:
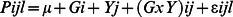
where *P_ij_* is the phenotypic value of genotype *i* for year *j*, *µ* is the overall mean of the progeny for all years and genotypes, *G_i_* is the random effect of genotype *i*, *Y_j_* is the fixed effect of the year *j*, *(GxY)_ij_* is their random interaction and, *ε_ijl_* is the random residual error effect for the *l* measured GUs per tree or trees per genotype.

For traits showing heterogeneous variances between years, a function of variance was introduced into the model. Different variances functions, as described by Pinheiro and Bates [33] were compared: i.e. varIdent, with different variances per level of the fixed factor; varPower, with variance increasing as a power function; varExp, with variance increasing as an exponential function; varConstPower, combines a constant value with a power function.

Moreover, because correlations between successive years may exist due to irregular and/or biennial bearing, a covariance structure was taken into account in the residual term of the corresponding mixed linear model. Different covariance structures were tested i.e. compound symmetry (corComSymm), autoregressive of order 1 (corAR1) and exponential (corExp). The minimization of Akaike and Bayes Schwarz information criteria (AIC and BIC respectively), allowed us to select the significant factors, variance function and covariance structure to be considered in the model. Therefore, the normality of residual distribution and model predictions adequacy with phenotypic values were checked.

All statistical analyses were performed using R software v.2.9.2, with REML estimation method, under lme4 and nlme packages [34].

Finally, heritability of flowering and fruiting genotypic means was estimated as the ratio between the genotypic and the phenotypic variances: H^2^ *=  σ^2^_G_/σ^2^_P_*. When significant interaction between the genotype and the year factor was selected, the heritability calculation was: H^2^ *=  σ^2^_G_/*[*σ^2^_G_*+*σ^2^_GxY_/a+σ^2^_ε_/na*] where *σ* ^2^ *_G_* is the genotypic variance, *σ^2^_GxY_* is the variance of genotype and year interaction, *σ^2^_ε_* is the residual error variance estimated from the selected model, *n* the number of replicates per genotype, and *a* the number of studied years.

When no significant interaction between the genotype and the year factor was selected, heritability was calculated as: H^2^ *=  σ^2^_G_/*[*σ^2^_G_*+(*σ^2^_ε_/n*)], where *σ^2^_G_* is the genotypic variance, *σ^2^_ε_* is the residual error variance estimated from the selected model, and *n* the number of replicate per genotype [35], [36].

### Linkage Map Construction

New genomic and expressed sequence tag (EST)-derived microsatellite (simple sequence repeat: SSRs) markers have been developed in olive and used to screen 147 genotypes from the ‘Olivière’ and ‘Arbequina’ progeny and the parents [Essalouh et al. unpublished data]. Polymorphic SSRs were integrated together with amplified fragment length polymorphism (AFLP), intersimple sequence repeat (ISSR) and SSR markers previously used in Khadari et al. [37], to build a new genetic map.

The Map construction was carried out using JoinMap version 4.0 [38]. Chi square values were calculated for each marker to detect segregation deviations (P≤0.05) from the expected Mendelian ratio. Highly distorted markers were discarded. Linkage groups (LG) were determined by a regression mapping procedure using an independence test of the logarithm of the odds (LOD score) with a minimum threshold of 5.0 [39]. Map construction was performed using the Kosambi mapping function [40] and the regression mapping algorithm following the JoinMap parameters: Rec = 0.40, LOD = 1.0, Jump = 5. The obtained LGs were drawn using the MapChart software [41]. New connections were established between the original LGs, in particular the smallest one. Likewise, the new intercross markers allowed connecting the parental LGs. Thus, LG numbering was redefined in the present maps and was defined as OA, O and A for the integrated map ‘Olivière’×’Arbequina’, the ‘Olivière’ female parent map and ’Arbequina’ male parent map, respectively.

To estimate observed genome coverage, the expected genome length in centiMorgans was estimated for each LG using the following method proposed by [42]: LG *expected length* = LG *observed length* * [(*m*+1)/(*m*-1)] where *m* is the number of markers of each linkage group. Observed genome coverage was assessed by dividing the observed genome length by the expected genome length.

### Quantitative Trait Loci (QTL) Detection

For each trait, the “Best linear unbiased predictions” (BLUPs) of random effects were extracted from the selected mixed linear models i.e. the genotype effect and used for QTL detection. The BLUP for the genotype effect was noted by the trait name. When significant, the BLUPs for the GxY factor were computed for each studied year of growth. These BLUPs were noted by the name of the trait followed by the considered year of growth.

Both new parental and integrated maps were used to identify QTL controlling flowering and fruiting traits at GUs scale as well as whole tree scale. QTL analyses were carried out using MapQTL version 6.0 [43]. A first step was the Kruskal-Wallis analysis in which, the effect of the marker significance level on a specific trait was estimated by simple linear regression between the marker and the trait [44]. Then, QTL mapping was performed using interval mapping (IM) which estimates the likelihood score of a putative QTL placed in any position within an interval flanked by two adjacent markers [45]. The LOD score threshold (P-value of 0.05) at which a QTL was declared significant, was determined using a permutation test for each LG as well as for the genome wide. Over 1000 permutations the frequency distribution of the maximum LOD score under the null hypothesis (no QTL) was determined [46]. When several markers displayed a LOD score equals or superior to the LOD score determined by the permutation test, they were declared as cofactors for a multiple QTL mapping (MQM) analysis with a step size of 1 cM [45].

Significant QTLs were characterized by their closest associated marker cofactors, LOD score, percentage of explained phenotypic variation, and confidence interval in centimorgans (cM) estimated on the basis of a LOD score drop of 1 or 2 on both sides of the likelihood peak. Genomic positions of the QTLs detected on the linkage groups were drawn using MapChart software [42]. Allelic effects were estimated as A_f = _[(µ_ac+_µ_ad_)_−_(µ_bc+_µ_bd_)]/4 for female additivity; A_m = _ [(µ_ac+_µ_bc_)_−_(µ_ad+_µ_bd_)]/4 for male additivity and D_ = _ [(µ_ac+_µ_bd_)_−_(µ_ad+_µ_bc_)]/4 for dominance where µ_ac,_ µ_ad,_ µ_bc_ _and_ µ_bd_ are estimated phenotypic means associated to each of the 4 possible genotypic classes *ac*, *bc*, *ad* and *bd*, deriving for an *ab*×*cd* cross [47], [48].

For traits showing multiple QTLs, a global model was built including all cofactors and their interactions in order to test epistatic effects between QTLs. A model selection was performed according to the Akaike’s information criterion (AIC) and Schwarz’s Bayesian criterion (BIC) minimization. The analysis was performed by a step model (i.e. iterative removal of non significant cofactors) using R software v.2.9.3, with REML estimation method, under nlme package [34].

## Results

In our study, the two parents ‘Olivière’ and ‘Arbequina’, differed significantly for flowering and fruiting traits and transgressive segregation was observed in the progeny (Table S1). The examination of production averages over four years showed that ‘Arbequina’ production was stable during the two first years then decreased in the following years whereas ‘Olivière’ showed an alternate bearing habit (Figure S2). The average production of the progenies also showed an alternate bearing with heavy productions in 2009 and 2011 (i.e. ‘ON’ year) whereas a lower production was observed in 2010 (“OFF” year”, Figure S1).

### Identifying Types of Bearing Behaviours in ‘Olivière’×‘Arbequina’ Progeny

**Figure 1:**
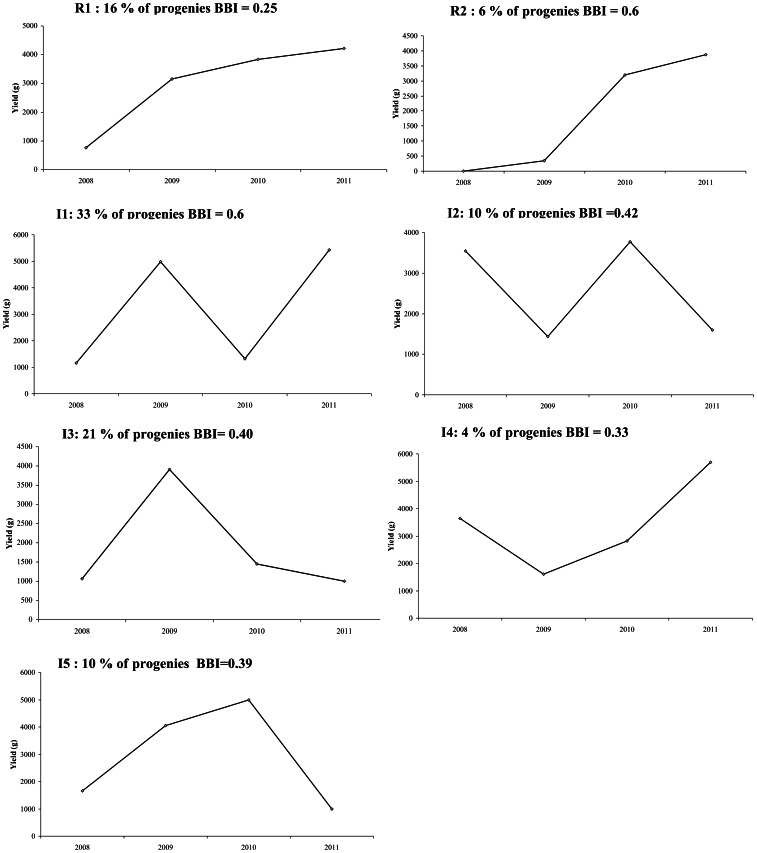
Seven bearing behaviours identified among genotypes within ‘Olivière’×‘Arbequina’ progeny based on the average Yield value per year and genotype. Progenies proportion within each class and average BBI value are indicated: BBI calculation takes into account the intensity of deviation in yield during successive years whatever its sign. Hence, similar BBI values were found for R2 and I2 class.

The examination of the sequences of the mean yield value per genotype over the four production years revealed seven bearing behaviours that were graphically identified in the population (Figure 1). Only 22% of the progenies exhibited a regular bearing behaviour (R1 and R2; Figure 1) among which 16% increased regularly their production during the four consecutive years and showed a BBI of 0.25 (R1; Figure 1). About 6% of these regular bearing progenies showed late production behavior since their yield did not exceed 1 Kg on average during the first 3 years of production and then increased in 2011 up to 2.7 Kg (R2; Figure 1). Considering these deviation, the R2 class showed a BBI of 0.6 (Figure 1). The remaining 78% of the progenies exhibited irregular bearing behavior (classes I1 to I5; Figure 1). Among this fraction, 43% were typically biennial bearing, but with two subclasses exhibiting opposite phases (33% and 10% for I1 and I2, respectively; Figure 1). Indeed, 2009 and 2011 were ‘ON’ years in I1 type whereas they were ‘OFF’ in I2. The BBI value was of 0.6 and 0.42 for I1 and I2, respectively (Figure 1). The last three types corresponded to progenies with irregular bearing. In I3 (21%), progenies were characterized by only one ‘ON’ year during the 4 studied years, that coincided with the ‘ON’ year of I1 (2009). In I4 (4%), progenies were characterized by a weak production in 2009 (as progenies in I2), followed by a constant increase in production reaching a yield of 6 Kg on average in 2011. In I5 (10%), progenies showed an increasing production from 2008 to 2010, as in R1, but this was followed by a drastic diminution in 2011, reaching a lower yield value than in 2008. The BBI values were of 0.4, 0.33, and 0.39 for I3, I4 and I5, respectively (Figure 1).

### Changes Over Time in the Number and Position of Inflorescences and Fruits Along GU

**Figure 2:**
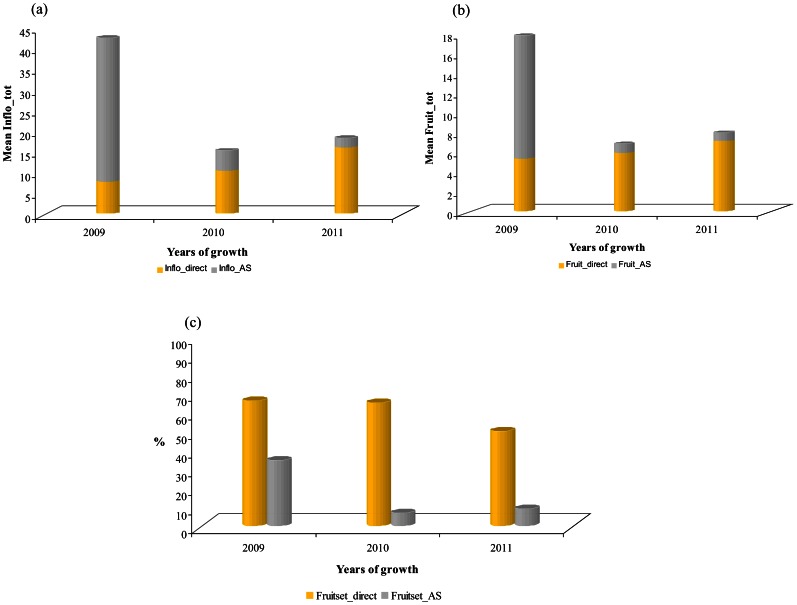
Illustration of the changes over time observed at flowering and fruiting periods on GUs in ‘Olivière’×‘Arbequina’ progeny: (a; b) Mean values of the number of inflorescences and fruits born along the leaf axils along GUs (Inflo(Fruit)_direct) and along GUs sylleptic laterals (Inflo (Fruit)_AS) as a function of years of growth (2009–2011); (c) Percentage of fruitset along the leaf axils along GUs and along GUs sylleptic laterals as a function of years of growth (2009–2011).

**Figure 3:**
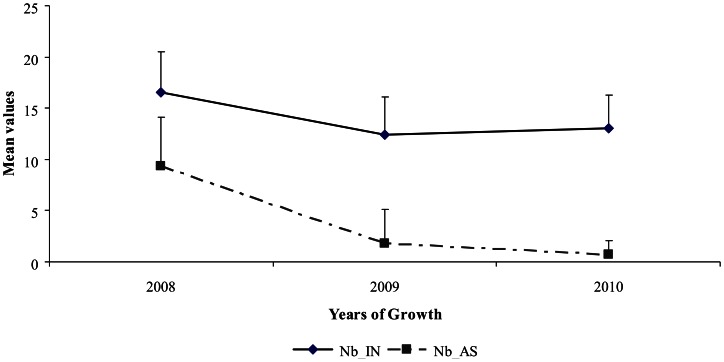
Mean values and standard deviations of the number of internodes and the number of sylleptic laterals of bearing floral GUs as a function of years of growth in ‘Olivière’×‘Arbequina’ progeny (2008–2010).

**Figure 4:**
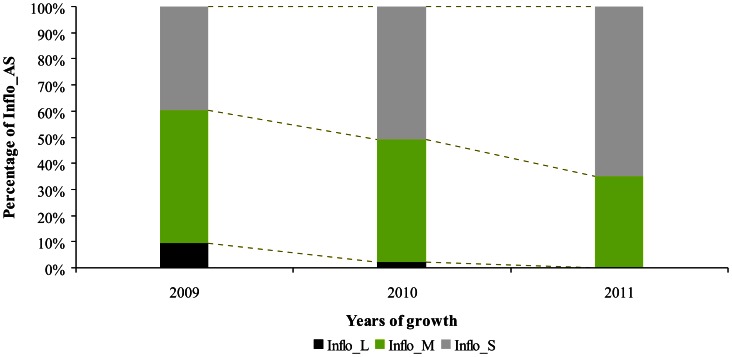
Percentage of inflorescences born along GUs sylleptic laterals types (long. **medium or short) depending on the year of growth in ‘Olivière’×‘Arbequina’ progeny.** Inflorescences on sylleptic laterals (Inflo_AS) were mainly born on short and medium types (Inflo_S and Inflo_M, respectively) in each studied year (2009–2011).

An important variability in both the number of inflorescences and their repartition along the one year-old GUs was found among the three studied years. In 2009, 42 inflorescences on average were observed, this number including inflorescences born along the GUs and their sylleptic laterals. In 2010 and 2011, the mean values were lower, 15 and 18 inflorescences respectively (Figure 2; a). This decrease was also observed for the number of fruits produced per GU which mean values ranged from 17.89 in 2009 to 6.88 in 2010 and 8 in 2011 (Figure 2; b). The number of inflorescences born directly along GUs (i.e. Inflo_direct) showed an increase from the first to the third year whereas a drastic reduction of the number of those born on GUs sylleptic laterals was observed. This was concomitant with the decrease in the number of GUs laterals (i.e. Nb_AS). It may be noticed that the number of internodes of the floral GUs remained almost stable over the same years (Nb_IN, Figure 3). Consistently, a negative correlation between Inflo_direct and Nb_AS was observed during the three reproductive years (r =  −0.42, data not shown). Changes were also found in the proportion of inflorescences born on each type of lateral GUs (Figure 4). The proportion of inflorescences on long sylleptic laterals (Inflo_L) ranged from 9.47% in the first year (2009) and decreased to 2.18% and 0% in the second (2010) and third year (2011), respectively. The proportion of inflorescences born on medium laterals (Inflo_M) also decreased whereas those on short laterals increased (Inflo_M and Inflo_S, respectively). Finally, in all studied years, a majority of inflorescences were born on short and medium types.

Changes over years were also observed on both Fruit_direct and Fruit_AS (Figure 2; b), with a progressive increase of the Fruit_direct and a decrease in the mean number of Fruit_AS. By contrast, fruit set (i.e. Total_Fruitset), observed whatever the inflorescences location, was relatively high (53%, 62% and 48% in 2009, 2010 and 2011, respectively). Fruit set was greater on direct inflorescences than on inflorescences born on sylleptic laterals whatever the year (Figure 2; c).

### Genetic Analysis

#### Significance and variance estimation of genotype and year factors

The total yield per tree showed heterogeneous variances over years, whereas a weak correlation was found between consecutive years, for the whole progeny, according to Kendall’s tau coefficients (Table S2). A strong genetic determinism was identified with highly significant genotype and interaction between the genotype and year (GxY) effects (Table 2). Highly significant effect of the year of production was also identified.

**Table 2:** Effects in selected models for traits related to reproductive development in ‘Olivière’×‘Arbequina’ progeny.

Variables	Factors			Variance Function	Covariance structure	Variance estimates			H^2^
**Tree scale**	*G*	*Y*	*GxY*			*V_G_*	*V_G :Y_*	*V_R_*	
Yield	**	**	**	–	–	239,58	242,66	476,78	0.60
**GUs scale**									
Inflo_tot	.	**	*	–	–	0,04	0,13	0,30	0.30
Inflo_direct	*	**	**	–	–	0,22	0,23	0,81	0.52
Inflo_AS	NS	**	**	–	–	–	1,18	2,93	NS
Inflo_L	NS	*	*	–	–	–	0,07	0,93	NS
Inflo_M	*	**	**	–	–	0,09	0,57	2,43	0.32
Inflo_S	.	**	**	–	–	0,03	0,47	1,65	0.36
Fruit_tot	**	**	NS	varExp	corExp	0.79	–	0.50	0.75
Fruit_direct	**	**	NS	varPower	corExp	0.67	–	1.00	0.57
Fruit_AS	NS	*	**	–	–	–	0,79	1,48	NS
Total_Fruitset	**	**	NS	varPower	corExp	6.81	–	2.28	0.85
Fruitset_direct	**	*	NS	varExp	corExp	6.50	–	5.57	0.70
Fruitset_AS	*	*	NS	varExp	–	0.49	–	3.27	0.23

Significance of effects: ns, not significant; *, significant (0.01<p≤0.05); **, highly significant (p≤0.01). Variance function and correlation structure when selected are indicated. Traits heritability (H^2^) was calculated as the ratio between genotypic and phenotypic variances estimates.

No significant correlation was observed between successive years for all flowering traits at GU scale, and models selected according to BIC criteria did not include any variance function (Table 2). The year factor was significant for all flowering traits whereas the genotype factor was significant only for two of them: Inflo_direct and Inflo_M. Highly significant interaction was found between the genotype and year factors for all these traits (Table 2).

Correlations between years were significant for all fruiting traits at GU scale, except Fruit_AS and Fruitset_AS, and an exponential spatial correlation structure corExp was selected in the corresponding models (Table 2). Heterogeneous variances were also found for all fruiting traits, again with the exception Fruit_AS, and were modeled by a power of covariate variance function (i.e. varPower; Table 2). Significant effects of both the genotype and year factors were found without significant interaction for all fruiting traits, except Fruit_AS. For this trait, genotype factor was not significant whereas interaction effect (GxY) was highly significant (Table 2).

#### Heritability of genotype mean

Heritability of genotype mean was estimated for all flowering and fruiting traits that showed significant genetic effect. Moderate heritability values were found for flowering traits, with H^2^ values ranging from 0.20 to 0.52 for Inflo_tot and Inflo_direct, respectively (Table 2). Considering fruiting traits, moderate to high heritability values were estimated with values exceeding 0.7 for Total_Fruitset and Fruitset_direct (Table 2). Likewise, a high heritability value was found for tree yield (H^2^ = 0.6).

#### Correlation between variables

Correlations between reproductive traits were investigated on the basis of the mean phenotypic values per genotype and year (Table S3). High Pearson correlation coefficients (r) were found between flowering and fruiting traits measured at GU scale: the total number of inflorescences (i.e. Inflo_tot) was highly correlated to the number of inflorescences born at the leaf axil along the GU (i.e. Inflo_direct) as well as the number of inflorescences born on GU laterals (i.e. Inflo_AS) in the three studied years (r values in italic; Table S3a). The correlation between Inflo_tot and Inflo_AS decreased over years (from 0.96 in 2009 to 0.7 in 2011) whereas that with Inflo_direct increased (from 0.42 in 2009 to 0.74 in 2011), consistently with the respective changes in these latter variables over years. Total fruit set was highly correlated to Fruitset_direct whatever the year (underlined r values; Table S3a). A slight negative correlation was found between the total number of inflorescences and the total fruit set in the three years with (−0.31 and −0.38 framed values; Table S.3a). The tree production was not or weakly correlated to flowering and fruiting trait at GU scale (Table S3b).

### Map Construction

In the present study, 94 new olive SSR were developed, of which 40 were from olive EST and 54 were from olive genomic library. SSRs were named ‘Zit’ referring to the Arabic word for ‘Oil’. EST-SSRs were designated with indexes from 001 to 113 following the name ‘Zit’ i.e. Zit001 while genomic-SSRs were designated with indexes from 302 to 505 i.e. Zit302. Among these 94 SSRs, 55 were polymorphic in both parents (Table 3). The remaining 39 SSRs were polymorphic in one parent only: 30 SSRs were polymorphic in ‘Olivière’ and 9 SSRs were polymorphic in ‘Arbequina’ (Table 3). Among these new SSRs, 53 (56%) were mapped on ‘Olivière’ female parent, 48 (51%) on ‘Arbequina’ male parent, and 72 (76%) on the integrated map ‘Olivière’×‘Arbequina’ (Table 4).

**Table 3:** Percentages of mapped SSRs ‘Zit’ in parental maps and integrated map.

Total Polymorphic SSRs	94		
*Maps*	*Zit*	*Mapped Zit*	*% total Zit*
‘Oliviere’	85	53	56%
‘Arbequina’	64	48	51%
Integrated	94	72	76%

**Table 4:** Segregation types identified among genomic and EST SSRs ‘Zit’ and their mapping percentage on integrated map.

Number of SSRs ZIT	Segegation	Genomic (*% mapped* *)	EST (*% mapped* *)
20	<abxcd>	11 (*90,9%*)	9 (*100%*)
35	<efxeg>	18 (*66,66%*)	17 (*70,58%*)
9	<nnxnp>	4 (*50%*)	5 (*60%*)
30	<lmxll>	21 (*76,19%*)	9 (*66,66%*)

* (% mapped on the integrated map).

The linkage analysis in ‘Olivière’ female parent was based on 362 markers including 257 AFLPs, 102 SSRs and 4 ISSRs. Among them, 212 markers including 68 SSRs were mapped in a total of 25 LGs (Table 5; Figure S3a). The ‘Olivière’ map length was 1745.3 cM, representing an observed genome coverage of 77.9%. The average marker spacing was 8.23 cM, with a maximum gap of 32.5 cM between adjacent markers observed in the LG O9 (Table 5, Figure S2a).

**Table 5:** Mapping characteristics of ‘Olivière’ female parent map, ‘Arbequina’ male parent map and ‘Olivière’×‘Arbequina’ integrated map: correspondence with the first genetic map characteristics are indicated between brackets.

Maps	LG(no.)	Mapped Markers(no.)	SSRs(no.)	Mean LGsize (cM)	Map Length (cM)	Average markerspacing (cM)	MaximumGap	Observed genomecoverage (%)
‘Olivière’	25 (34)	212 (197)	68 (34)	69.81 (61.3)	1745.3 (2210.2)	8,23 (11.2)	32,5 (48.5)	77,9
‘Arbequina’	21 (31)	252 (191)	75 (30)	76.07 (63.4)	1597.6 (1966.2)	6,34 (10.3)	30,8 (40.4)	83,8
Integrated	26 (42)	450 (436)	103(26)	82.63 (91.0)	2148.4 (3823.2)	4,77 (8.7)	30,5 (81)	86,9

Correspondences with the first genetic map characteristics are indicated between brackets.

The linkage analysis in ‘Arbequina’ male parent was based on 352 markers including 256 AFLPs, 89 SSRs and 7 ISSRs. Among them, 252 markers including 75 SSRs were mapped in a total of 21 LGs (Table 5; Figure S3b). The ‘Arbequina’ map length was 1597.6 cM, representing an observed genome coverage of 83.8%. The average marker spacing was of 6.34 cM with a maximum gap of 30.8 cM between adjacent markers observed in the LG A14 (Table 5, Figure S3b).

**Figure 5:**
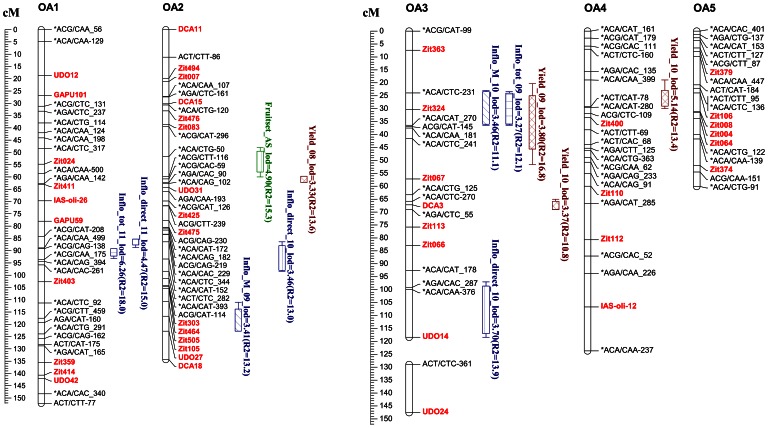
Genomic positions of the QTLs detected on the linkage groups of the ‘Olivière’×‘Arbequina’ integrated map by multiple QTL mapping (MQM) for the best linear unbiased predictors (BLUPs) of reproductive traits measured at both tree and GUs scale. Map distances were derived using the Kosambi mapping function and SSRs markers were colored in red. QTLs are represented by boxes extended by lines representing the LOD-1 and LOD-2 confidence intervals. Boxes are coloured according to the process of the traits: Blue and Green for flowering and fruiting at GUs scale, respectively; Brown for production at whole tree scale. For each trait, a distinct fill style was used for boxes representing QTLs. For each QTL, corresponding BLUP (computed from genotype effect or from the interaction between genotype and year), LOD peak and R^2^ were indicated as detailed in Table 6.

Among the 535 segregating markers (407 AFLPs, 117SSRs and 11 ISSRs), 450 were mapped in the ‘Olivière’×‘Arbequina’ integrated map including 103 SSRs (Table 5). The markers were assigned to 26 linkage groups (Table 5; Figure 5). The integrated linkage map was 2148.4 cM, representing an observed genome coverage of 86.9%. The average marker spacing was of 4.77 cM with a maximum gap of 30.5 cM between adjacent markers observed in the LG OA14 (Table 5).

### QTL Detection and Mapping

Results of QTL detection are detailed for each reproductive trait measured at both tree and GU scales. QTLs detected on the integrated map ‘Olivière’x‘Arbequina’ are presented in Table 6 and Figure 5. Those that were also detected on parental maps are indicated in bold in Table 6. QTLs detected on parental maps are detailed in the supporting information file (Table S4). In the following sections, QTLs for G effect BLUPs are described first. Second, results of QTL analysis for year-specific BLUPs are provided. In all cases, QTLs with the highest percentage of explained variability are detailed first, and co-localisations between QTLs are mentioned.

**Table 6:** QTLs detected on ‘Olivière’×‘Arbequina’ integrated map.

BLUP	Linkage Group	LOD^a^	Var (%)^b^	Allelic effect^c^	A_f_ c	A_m_ c	D^c^	Cofactor^d^
**Tree Scale**								
Yield	**OA12**	4.35 (2,5)	15.1	Am	−0,7267	4,8602	−0,8794	*Zit053*
								
Yield_08	**OA2**	3.33 (3.4)	13.6	Af	−3,0397	1,1436	1,1755	*ACG/CAC-59
	**OA6**	2.39 (2.4)	9.6	Af;D	2,6439	−0,3025	−1,4886	Zit482
Yield_09	**OA3**	3,80 (3,0)	16.8	Af; Am	3,4688	1,1943	0,3547	*Zit324*
Yield_10	OA4	5.14 (3,1)	13.4	Af; D; Am	2,4808	−2,5112	−1,8372	*ACT/CAT-78
	OA9	3.45 (2,8)	11.1	D	0,6723	−0,7503	−3,1309	*AGA/CTG-117
	**OA3**	3.37 (2,9)	10.8	Am; D	−0,9899	2,9788	1,2178	*DCA3*
Yield_11	**OA25**	4.50 (2,8)	20.2	Af; D; Am	−5,284	4,008	−2,909	Zit388
**GU Scale**								
Inflo_tot	**OA25**	3,45 (2,6)	11.6	Am	0,0039	−0,0402	0,0156	**Zit342**
Inflotot_09	OA25	4.00 (2,8)	14.7	D	0,0370	−0,0324	0,1217	**Zit342**
	**OA3**	3.27 (3,0)	12.1	Am	0,0335	0,1062	0,0231	*Zit324*
Inflotot_10	**OA13**	4.11 (3,4)	16.6	Am	−0,0243	0,1241	0,0169	*Zit376*
Inflotot_11	**OA1**	**6.26** (3,3)	18.0	Am; D	−0,0045	−0,0605	0,0570	*AGA/CAA_142
	OA7	**5.06** (2,3)	14.3	Af; Am	0,0500	−0,0457	−0,0226	*ACT/CTT_197
Inflo_direct	**OA8**	3,33 (3,0)	12,1	Am	0,0707	0,1156	0,0488	*ACT/CAC_142
Inflodirect_09	**OA16**	4.01 (3,2)	15	Af	−0,1519	−0,0556	0,0482	*ACG/CTC-128
Inflodirect_10	OA3	3.70 (2,9)	13.9	Af; Am	−0,0782	−0,0827	0,0421	*ACA/CAA-376
	**OA9**	3.47 (2,7)	9.5	Af; D	−0,0694	0,0097	−0,0609	*AGA/CTG-117
	OA2	3.46 (3,2)	13.0	Af; D; Am	−0,0446	−0,0651	−0,0784	*ACA/CAT-152
Inflodirect_11	OA1	4.47 (3,3)	15.0	Am; D	0,0151	−0,0471	0,0750	*AGA/CAA_142
	**OA9**	3.64 (2,7)	12.0	Af	0,0800	−0,0139	−0,0223	*AGA/CTC-83
	**OA16**	3.03 (3,0)	9.8	Af	0,0652	−0,0074	0,0151	*ACG/CTT-326
Fruit_direct	**OA20**	3.12 (2,8)	13.1	Af; Am	0,1186	0,2819	−0,0169	*AGA/CTT_143
Fruitset_AS	**OA2**	**4.90** (3.3)	15.3	Af; D	−0,2623	0,1104	−0,1618	*ACA/CTG-50
Inflo_M_09	**OA2**	3.48 (3,4)	13.2	Af; Am	0,2260	−0,1695	−0,0955	Zit105
Inflo_M_10	**OA3**	3.46 (2,9)	11.1	Am	−0,0084	−0,1079	0,0145	*Zit324*
Inflo_M_11	**OA20**	3.71 (3,0)	13.3	Am	0,0360	0,0698	0,0342	*ACA/CTG_330
	OA7	3.47 (2,3)	12.4	Af; D; Am	0,0366	−0,0387	−0,0519	**Zit447**
Inflo_S	OA8	**5.57** (2,9)	19.1	Af; D	−0,0082	0,0013	0,0225	Zit394
	**OA10**	4.19 (3,0)	14.1	Af	0,0202	0,0009	−0,0025	Zit402
	OA9	2.64 (2.6)	8.4	Af; D	−0,0088	0,0019	0,0140	*AGA/CTG-117
Inflo_S_09	OA20	**4.61** (2,9)	12.9	Af; D	−0,1697	−0,0416	−0,1447	**Zit417**
	OA11	3.08 (3.0)	8.3	D	0,1014	0,0961	0,1415	*Zit309*
	**OA21**	3.08 (3,1)	8.3	Am; D	−0,0122	−0,1788	−0,0891	**IAS_oli_11**
Inflo_S_11	**OA7**	3.35 (2,4)	10.1	Am	0,0335	−0,1024	−0,0333	**Zit447**

a Maximum LOD score value with the considered threshold in parentheses: Bold LOD score values are significant at genome wide threshold.

b Percentage of phenotypic variation explained by the QTL.

c Allelic effects were estimated as A_f = _[(µ_ac+_µ_ad_) − (µ_bc+_µ_bd_)]/4 for female additivity; A_m = _ [(µ_ac+_µ_bc_)_−_ (µ_ad+_µ_bd_)]/4 for male additivity and D_ = _ [(µ_ac+_µ_bd_) − (µ_ad+_µ_bc_)]/4 for dominance where µ_ac,_ µ_ad,_ µ_bc_ _and_ µ_bd_ are estimated phenotypic means associated to each of the 4 possible genotypic classes *ac*, *bc*, *ad* and *bd*, deriving for an *ab*×*cd* cross.

d markers used as cofactors in the MQM analysis: Bold cofactors are mapped on ‘Arbequina’ genetic map (male parent); Non Bold cofactors are mapped on ‘Olivière’ genetic map (female parent) and Italic cofactors are mapped on both parental maps.

#### Reproductive traits measured at tree scale

A QTL explaining 15.1% of the variability was detected on OA12 for yield per tree (Table 6; Figure 5). Seven others QTLs were detected for year-specific BLUPs of Yield. Two were detected on OA2 and OA6 for yield in 2008 and explained 13.6% and 9.6% of the variability, respectively (Yield_08; Table 6, Figure 5). Both QTLs were mainly due to a female additive effect. A global model built for these QTLs showed no significant interaction between cofactors and explained 16.4% of the trait variability (Table 7).

**Table 7:** Global model estimations for reproductive traits with several QTLs detected.

BLUP	Linkage Group	Cofactor^a^	p-value^b^	Global R^2^ c
**Tree scale**				
Yield_08	OA2	*ACG/CAC-59	0.0002	0.164
	OA6	Zit482		
Yield_10	OA4	*ACT/CAT-78	0.002	0.188
	OA9	*AGA/CTG-117		
	OA3	*DCA3*		
**GU Scale**				
Inflotot_09	OA25	Zit342	0.0001	0.244
	OA3	*Zit324*		
Inflotot_11	OA1	*AGA/CAA_142	3.266e-05	0.190
	OA7	*ACT/CTT_197		
Inflodirect_10	OA3	*ACA/CAA-376	0.001	0.143
	OA9	*AGA/CTG-117		
	OA2	*ACA/CAT-152		
Inflodirect_11	OA1	*AGA/CAA_142	1.665e-05	0.225
	OA9	*AGA/CTC-83		
	OA16	*ACG/CTT-326		
Inflo_M_11	OA20	*ACA/CTG_330	2.728e-05	0.237
	OA7	Zit447		
Inflo_S	OA8	Zit394	8.959e-06	0.2563
	OA10	Zit402		
	OA9	*AGA/CTG-117		
Inflo_S_09	OA20	Zit417	2.875e-06	0.402
	OA11	*Zit309*		
	OA21	IAS_oli_11		

a Effects selected according to AIC and BIC minimization.

b Effect probability.

c Percentage of variation explained by the global model.

One QTL was detected on OA3 for the yield in 2009 (Yield_09; Table 6, Figure 5). This QTL explained 16.8% of the variability and resulted from both female and male additive effects. Three QTLs were mapped on OA4, OA9 and OA3 for the yield in 2010 (Yield_10; Table 6, Figure 5). These QTLs explained 13.4%, 11.1% and 10.8% of the variability, respectively. The QTL mapped on the median part of OA3 resulted from both male additive and dominance effects. The QTL detected on OA4 resulted from female and male additive as well as dominance effects while the QTL detected on OA9 resulted from dominance effect (Table 6). Together, these QTLs explained 18.8% of the variability (Table 7). One QTL was detected on the distal part of OA25 for the yield in 2011 (Yield_11; Table 6, Figure 5). This QTL explained 20.2% of the variability and resulted from both female and male additive as well as dominance effects.

#### Reproductive traits measured at GU scale

A QTL was detected on the median part of OA25 for the total number of inflorescences per GU (Inflo_tot; Table 6, Figure 5). This QTL explained 11.6% of the variability and resulted from male additive effect (Table 6). Five QTLs were found for year-specific BLUPs for this variable. The first QTL detected for 2009 co-localized with the QTL previously detected for Inflo_tot on OA25 (Inflotot_09; Figure 5). This QTL explained 14.7% of the variability and resulted from a dominance effect (Inflotot_09; Table 6). A second 2009 specific QTL was found on OA3 and co-localized with the QTL previously found for Yield_09 (Figure 5). This QTL explained 12.1% of the variability and resulted mainly from a male additive effect (Table 6). The global model did not include any interaction between QTLs and explained 24.4% of the variability (Table 7).

Considering the 2010 BLUPs, a QTL was identified on the distal part of OA12 and explained 16.6% of the variability. This QTL resulted from male additive effect (Inflotot_10; Table 6). Two 2011 specific QTLs were detected on OA1 and OA7. The QTL mapped on the median part of OA1 explained 18.0% of the variability and resulted from both male additive and dominance effects (Table 6). The QTL on the distal part of OA7 explained 14.2% of the variability and resulted from both female and male additive effects (Table 6). The global model did not include any interaction and explained 19.0% of the variability (Table 7).

A QTL was detected on the median part of OA8 for the number of direct inflorescences per GU (Inflo_direct; Table 6, Figure 5). It explained 12.1% of the variability and resulted from male additive effect (Table 6).

Seven other QTLs were detected for year-specific BLUPs of Inflo_direct. One QTL detected on OA16 for Inflo_direct in 2009 (Inflodirect_09; Table 6, Figure 5) explained 15.0% of the variability and resulted from female additive effect. Three 2010 specific QTLs were detected on OA3, OA9 and OA2 (Inflodirect_10, Table 6). The first QTL on the distal part of OA2 explained 13.9% of the variability and resulted from both female and male additive effects (Table 6). The second QTL on OA9 co-localized with a QTL previously detected for yield in 2010 (Figure 5). This QTL explained 9.5% of the variability and resulted from both female additive and dominance effects (Table 6). The third QTL detected on the distal part of OA2 explained 13.0% of the variability and resulted from both female and male additive as well as dominance effects (Table 6). Together the three QTLs explained 14.3% of the Inflo_direct variability (Inflodirect_10; Table 7).

Three QTLs were detected on OA1, OA9 and OA16 for 2011 BLUPs of Inflo_direct (Inflodirect_11; Table 6, Figure 5). The first on OA1 co-localized with a QTL previously detected for the 2011 BLUP of Inflo_tot (Figure 5). It explained 15.0% of the variability and resulted from male additive and dominance effects (Table 6). The QTLs detected on OA9 and OA16 explained 12% and 9.8% of the variability and resulted from female additive effect (Table 6). Altogether these QTLs explained 22.5% of the variability (Inflodirect_11; Table 7).

QTLs were detected for genotype effect BLUP of the number of inflorescences per short laterals only (Inflo_S). Three QTLs were detected on OA8, OA10 and OA9 (Table 6, Figure 5), and explained 19.1%, 14.1% and 8.4% of the variability, respectively. The QTL mapped on OA8 co-localized with the QTL previously detected for Inflo_direct whereas that mapped on OA9 co-localized with both the previously detected QTLs for yield in 2010 and Inflo_direct (Figure 5). Both QTLs resulted mainly from female and dominance effects. The third QTL mapped on the distal part of OA10 resulted from female additive effect (Table 6). The global model did not include any interaction and QTLs explained 25.6% of the variability (Inflo_S; Table 7).

Year-specific QTLs were detected for both BLUPs of Inflo_M and Inflo_S. Four QTLs were detected for the year-specific BLUPs of Inflo_M. One was detected on OA2 for Inflo_M in 2009 (Inflo_M_09; Table 6, Figure 5), which explained 13.2% of the variability and was mainly due to female additive effect. A QTL was detected on OA3 for the 2010 BLUPs. This QTL explained 11.1% of the variability and resulted mainly from male additive effect (Inflo_M_10; Table 6, Figure 5). Two QTLs were detected on the distal part of OA20 and OA7 for the 2011 BLUPs, and explained 13.3% and 12.4% of the variability, respectively (Inflo_M_11; Table 6, Figure 5). The QTL identified on OA7 co-localized with the QTL previously detected for Inflo_tot in the same year (Figure 5) and resulted from both female and male additive as well as dominance effects whereas that on OA20 resulted from male additive effect (Table 6). Together, these QTLs explained 23.7% of the variability (Inflo_M_11, Table 7).

Three 2009 specific QTLs were detected on OA20, OA11 and OA21 for BLUP of Inflo_S (Inflo_S_09; Table 6, Figure 5). The QTL mapped on the medium part of OA20 explained 12.9% of the variability and resulted from both male additive and dominance effects. The QTL on OA11 explained 8.3% of the variance and was mainly due to dominance effect. The QTL on OA21 explained 8.3% of the variance and resulted from both male additive and dominance effects. Together, the three QTLs explained 40.2% of the variability (Inflo_S_09, Table 7). One 2011 specific QTL detected on OA7 (Inflo_S_11; Table 6, Figure 5) explained 10.1% of the variability and resulted from male additive effect (Table 6).

A QTL was detected on OA20 for the number of direct fruits per GU (Fruit_direct; Table 6, Figure 5). This QTL explained 13.1% of the variability and mainly resulted from male additive effect (Table 6). Another QTL was detected on OA2 for the percentage of axillary fruit set (Fruitset_AS; Table 6, Figure 5). It explained 15.3% of the variability and resulted from female additive and dominance effects (Table 6).

## Discussion

### Fruiting Behaviour of Progenies, Quantification and Scale of Description

For most alternate bearing species, alternate bearing phenomenon is linked to a lack of flower bud differentiation, commonly observed following a heavy fruiting year [5], [8]. Considering the large population size and the intense flowering and abscission characteristic of olive, it was not realistic to quantify the total number of inflorescences per tree, as previously proposed by Guitton et al. [8]. Rather we chose to quantify the total yield per tree and year, and to describe a sub-sample of one-year old GUs, at flowering and fruiting stages. Based on yield per year, genotypes could be classified according to their bearing pattern. The evaluation of ‘Olivière’×‘Arbequina’ population over the first four production years showed the segregation of fruiting behaviour among progenies with approximately 3/4 of irregular or alternate bearing progenies (78%) and 1/4 of regular progenies (22%). We thus consider that yield and its variation depending on the years is suitable for characterising the fruiting behaviour in an olive tree progeny. Each class of trees with contrasted patterns of fruiting behaviour was characterised by its mean biennial bearing index (BBI) [11]. However, since BBI calculation takes into account the intensity of deviation in yield during successive years whatever its sign (i.e. absolute value), its values did not correctly captured the differences between trees categories as identified in Figure 1. These results are consistent with those observed on apple [8]. In fact, BBI has been reported as being efficient for characterising a strictly biennial pattern but to a less extent a triennial one [49]. The same authors also recommended to evaluating BBI with series of at least 10 years, and when the production is stabilised. Indeed, during the first years of tree maturity, BBI is sensitive to the tree’s yield variation due to the ontogenetic increase in tree production in addition of the annual variation [50]. Because it may be of interest for selection schemes to be able to distinguish between regular and irregular bearing genotypes as soon as possible during tree ontogeny, these authors have proposed to remove trend due to the increase in fruit production in the first production years and accounting for year’s variations exclusively i.e. the between years residuals of *y*. To this end, an index based on deviation around yield trend over years and accounting for the dependencies between consecutive years was developed by Durand et al. [unpublished data]. This approach could notably improve biennial bearing estimation and could be further explored in olive progenies. Besides estimating the bearing irregularity through BBI, other indices could be considered as for instance the quantification of synchronism of flowering occurrences within branches or trees as previously proposed by Lauri et al. [51], [52]. In the present study, because the number of years was still low, we did not used BBI at genotype level, as a variable for genetic study and QTL detection.

By contrast, the variables characterising the flowering and fruiting at GU scale, did not capture the ‘ON’ or ‘OFF’ status of the tree in a given year. This can be exemplified by comparing the two ‘ON’ years: in spite of the lower amount of flowering in 2011 at GU scale, the final fruit production was almost similar to that found in 2009 when the flowering was the highest along GUs. Likewise, Fruit set variables were not impacted by the bearing status of the trees, e.g. the final fruit production in 2010, was low despite high fruit set (see Figure S2). This suggests that it is neither the amount of flowering nor the fruit set percentage along GUs determine the final fruit production of trees. Similar finding was reported in olive tree by Lavee [53] who underlined the lack of direct relationship between the production and the initial abundance in flowers at the tree scale. The lack of relationship between yield at tree scale and flowering and fruiting variables at GU indicate that our GU sampling strategy must be reconsidered. Indeed, we selected each year GUs that were similar in length and with at least some flowers. This choice may have introduced a bias since ‘OFF’ trees are likely to have a large number of shoots without any flowers which were avoided. Alternatively to our choice, sampling branched systems could be more appropriate in the objective to capture the tree bearing status in a given year and evaluate the synchronism of flowering occurrence within branches (see below).

### Effect of Climatic Years and Tree Ontogeny

The climatic year significantly impacted all studied traits. Considering that trees were regularly irrigated in our experiment and that inflorescences initiation is known to be insensitive to photoperiod in olive [54], [55], temperature constrains could be the main climatic effect that could affect flowering in our progeny (Figure S4). Regulation of flowering by temperature has been widely studied in olive tree [54]–[58]. Moreover, fertilization success is very sensitive to extreme temperature since it inhibits pollen germination and slow down or stop the pollen tube [59]. The receptivity of the stigma can also be shortened by dry winds and hot environmental conditions [60]. In addition, the co-existence in a given year of both ‘ON’ and ‘OFF’ trees (as it was the case in 2009 and 2011 which were ‘ON’ in average in the progeny, but with 16% and 41% of ‘OFF’, respectively), led us considering that climatic conditions in a given year are not directly responsible for tree bearing status. However, our experimental design did not allow us to distinguish between the ontogenetic and climatic effect within the year effect as proposed by Segura et al. [61]. Nevertheless, changes in flowering and fruiting potential at the GU scale could be interpreted in relation with their growth and branching, giving an insight into tree ontogeny. After a plentiful flowering and fruiting in 2009, the total number of inflorescences and fruits per GU decreased in the two following years (2010 and 2011). Because, in olive tree, inflorescences develop from the axillary buds of well lignified shoots induced the previous year [14], this decrease is due to that of sylleptic branching whereas the number of inflorescences and fruits located at the GUs leaf axil increased regularly. This is also supported by the negative correlation between the number of GUs laterals and the number of inflorescences born at GUs leaf axils. Here we show that this negative correlation, previously reported in Ben Sadok et al. [29], is maintained over the three studied years which correspond to the tree mature phase. Consistently with Rugini and Panelli [62], these results show an antagonism in the development of axillary meristem which can be either vegetative laterals or inflorescences born at the leaf axils along GUs. Likewise, the number of internodes per GU decreased slightly in the same period but this low reduction in the potential number of flowering sites was compensated by the reduction of sylleptic laterals, since the number of flowers born at GU leaf axils (or Inflo_direct) increased regularly overs the years. These observations can also be interpreted with respect to tree ontogeny. Indeed, we have shown a significant decrease in primary growth and sylleptic branching in the progeny during the first years of growth, reaching a stable phase at first flowering occurrence [29]. Here, we complement our analysis showing that this stable phase is maintained so far until 2010 and is characterised by a clear shift towards short shoots. This is consistent with studies previously carried out on fruit [63]–[66] and forest trees [67]–[69]. The predominance of short length shoots in mature trees has also been already reported in Hojiblanca olive cultivar in both bearing and non-bearing years [22]. However, it must be noticed that the ‘reiteration’ phenomenon which can give rise to long and vigorous branched systems, even late in the tree ontogeny [69]–[71], is frequent in olive tree. Indeed, this morphogenetic process, which was not investigated in the present study, is at the basis of “production unit” [19].

### A New Olive Genetic Map as an Efficient Tool for QTL Mapping

One of the targets of this study was to use the 94 new developed SSRs in olive [Essalouh et al. unpublished data] to enrich a genetic map previously constructed with AFLP, ISSR and few SSR markers [37]. Map saturation and LGs in the first maps were considerably improved by the addition of SSRs. As a result, 25 LGs covering a total of 1745.3 cM and 21 LGs covering a total of 1597.6 cM were obtained for the female ‘Olivière’ and male ‘Arbequina’ parental maps, respectively. Khadari et al. [37] reported 2210.2 cM for ‘Olivière’ and 1966.2 cM for ‘Arbequina’. Differences in genome length with the initial maps result mainly from the differences in the average distance between adjacent markers. The higher length of the female linkage map could indicate a higher recombination rate for the ‘Olivière’ female parent during meiosis as previously reported in various species such as Arabidopsis thaliana [72], tomato [73] and apple [74]. Using strict grouping parameters, we obtained a number of LGs close to the number of haploid chromosomes (i.e. n = 23) in which markers were well distributed. Similar results were obtained by Wu et al. [75] for ‘Frantoio’ and ‘Kalamata’ cultivars. Even though, these maps were based on a limited number of markers (i.e. 104), and were constructed with a more relaxed grouping parameters that those used in our study (i.e. recombination fraction = 0.49, LOD threshold = 3).

The integrated linkage map developed in the present study cover 2148.4 cM and is saturated at 86.9%. In comparison to previously developed maps in olive i.e. ‘Leccino’ and ‘Dolce agogia’ [76]; ‘Frantoio’×‘Kalamata’ [75]; ‘Picholine marocaine’×‘Picholine du Languedoc’ [77] and ‘Olivière’×‘Arbequina’ [37], the present integrated map ‘Olivière’×‘Arbequina’ includes the largest number of molecular markers i.e. 450 markers of which 103 are SSRs. The density of markers in the present map (i.e. one marker every 4.77 cM) is higher than that observed in the previous olive genetic maps (i.e. one marker every 10.2∼8.1 cM [75], [77]). Marker order in the integrated map was consistent with marker order in each parental map reflecting its precision.

### QTL Detection and BLUP Correlation

In the present study, a two-stage approach has been chosen whereby the best linear unbiased predictors (BLUPs) of the genotypic effect and its interaction with the year factor were obtained first from the mixed linear models of repeated data and then were used for interval mapping (IM) analysis and multiple QTL mapping (MQM) which allows reducing type I error (a QTL is indicated at a location where there is no QTL present) and type II error (a QTL is not detected) for QTL detection [45]. BLUPs of genotypic effect were thus considered as independent of ontogenetic and climatic effects, while BLUPs of interaction effects between genotype and year were assumed to be specific of a year. This method has been used in apple, optimizing the statistical power to detect significant QTL [48]. Nevertheless, such approach could lead to a higher false positive rate and inferences about the magnitude of QTL effects could be biased [78], [79]. One-step modelling of phenotypes in which genotype effect would be replaced by molecular marker effects could be considered to improve this method. In addition to the application of such new mapping procedure that requires high computational time, QTL analysis resolution could be improved through increases in sample size and marker number.

A strong genetic control was highlighted for most flowering and fruiting traits with relatively high heritability values, yet specific to the progeny under study and the climatic years analysed [80]. Most QTL were not significant at genome wide LOD threshold. This may be due to high LOD threshold value for some LG. Most QTLs showed small effects, suggesting that traits related to the reproductive development have a multigenic control as previously reported on apple tree [8]. The majority of QTL was associated to flowering traits, most of them being Year-specific QTL. Their higher number in comparison to those based on genotype effects BLUPs is consistent with the significant GxY effect. In addition, the QTLs detected for BLUP of genotypic effect for either yield or flowering and fruiting variables at GU scale were mainly under allelic effects from Arbequina parent. This is consistent with the high production potential [30], [81].

Two QTL were found for BLUP of genotype effect on Yield and Inflo_tot, but without co-localisations between them. The QTL for Yield (on OA12) had a main and positive effect coming from ‘Arbequina’, and associated to the allele of *Zit053* locus of size 150 bp. This QTL having a large effect needs to be further confirmed. Indeed, we cannot exclude the risk this QTL being a false positive because of the relative low size of our population. The significant statistic correlation between BLUPs of genotype effect on tree yield and the number of fruits and fruit set at GU scale suggests that these variables could have a common genetic control. However, no co-localization was found between the associated QTLs. The QTL found for BLUP of genotype effect on Inflo_tot (on OA25) co-localized with that detected for Inflo_tot_2009, consistently with the significant correlation found between these BLUPs (r = 0.54, see Table S5). This co-localisation can be interpreted as the result of the high variance for Inflo_tot observed in 2009 which was the first year of high flowering and was considered as ‘ON’ year.

Numerous QTLS were also detected for all variables either in a specific year or born on a specific position along GUs. No co-localization was found between the year-specific QTL for yield, this revealing the absence of a common genetic control. By contrast, co-localisations were found between year-specific QTLs for yields and either QTLs of total number of inflorescences or fruit set at GU scale. The QTL for yield in 2008 (on OA2) co-localized with that found for Fruitset_AS. For yield in 2009, the QTL detected on ‘Arbequina’ parental map only (on A23) co-localised with QTL associated with the three Fruitset variables (Total_Fruitset, Fruitset_direct and Fruitset_AS). It is noticeable that (i) these co-localisations do not rely on significant correlations between these variables (see Table S5) and (ii) no co-localisations were found in the last two years, 2010 and 2011, even though QTLs were detected for yield in both these years. Although revealed in the two first production years only, these co-localizations confirm the common genetic control between Fruit set and Yield. As the number of lateral flowering GUs was the highest during these two first years, we could assume that the tree production for a giving year is likely to rely more on branching, and therefore on the number of flowering GUs present within the trees, rather than on the number of inflorescences per GU, particularly when the trees are young. This highlight the interaction between the vegetative growth and branching with the reproductive behaviour reported at the whole tree in olive [19], [22].

In 2009 and 2010, the QTLs for Yield co-localized with QTLs detected for Inflo_tot_2009 (OA3) and inflo_direct_2010 (OA9), respectively. The co-localization in 2009 is consistent with the relatively high correlation between the two variables and can be interpreted as previously with respect to the high flowering observed in 2009. It is noticeable that the co-localisation in 2010 concerns the number of inflorescences born directly along GU. This may indicate that vegetative growth could have a higher impact on yield, when tree is ageing and its branching decreases.

Several co-locations were also highlighted between QTLs for the number of inflorescences in a specific year or born on a specific lateral type. Regarding genotypic BLUP of number of inflorescences in specific positions, three non-epistatic QTLs were mapped for Inflo_S. One of them overlapped the genomic region associated to the genotypic BLUP of Inflo_direct (on OA8). This could not be interpreted by their correlation, but rather by a common genetic control of flowering at local scale, probably at meristem scale. Another QTL for Inflo_S co-localized with Inflo_direct in 2010. In addition to co-localisations previously commented, QTLs for Inflo_tot, Inflo_M and Inflo_S in 2011 overlapped in a specific region of OA7. Another QTL found for Inflo_tot in 2011 was in the same genomic region as that for Inflo_direct in the same year (on OA1). These co-localizations are consistent with significant correlations between these traits. All together they indicate that flowers born either on short and medium laterals or directly along GUs, both contribute to the total number of flowers, but may involve different genomic regions depending on the year. Moreover, among all QTLs, the three QTLs for Inflo_S in 2009 explained the highest proportion of phenotypic variance. All QTLs detected for Inflo_S either for the genotypic BLUP or specific to 2009 confirm the large variation of the number of inflorescence born on short shoots in the studied olive tree progeny, and reveal a complex genetic determinism for this trait, associated to the most abundant branching type in mature olive trees [22].

### Conclusion

Our study of the olive reproductive behaviour has provided new knowledge about its complex genetic control. Progenies showing an increasing production over the first four years of mature phase and a regular bearing pattern constitute a promising material for olive breeding programs for regularity improvement. However, additional production years need to be further investigated in order to validate their stability over time. Two comprehensive maps for the cultivars ‘Olivière’ and ‘Arbequina’ and one integrated map ‘Olivière’×‘Arbequina’ were developed, including the largest number of SSRs so far. Additional SSR markers will be added in the future in order to expand the genome coverage and obtain more bridge markers that will be useful not only in saturating our maps but also for comparative mapping studies.

## Supporting Information

Figure S1 **Schematic representation of a 5-year-old olive tree phenotyped in 2009: flowering and fruiting traits collected on 1-year-old GUs at flowering and fruit set periods; the number of inflorescences and fruits born along the floral GUs (Inflo(Fruit)_direct) or along their sylleptic laterals (Inflo(Fruit)_AS) were counted.** Floral buds in year i (2009 in the present case) are induced during the summer of year i-1 (2008 in our case) and were thus born on GUs of year i-1. Their final differentiation occurs in year i [14].
(TIF)

Figure S2 **Mean Yield values and standard deviations (SD) for the parents ‘Olivière and ‘Arbequina’ and the progeny over four years (2008–2011).**
(TIF)

Figure S3 **Representation of parental genetic linkage maps (a) ‘Olivière’ female parent map (b) ‘Arbequina’ male parent map.** Map distances were derived using the Kosambi mapping function. AFLP markers presented without ‘*’are segregating in both parents. SSRs markers are colored in red
(TIF)

Figure S4 **Temperature records during 2009–2011: (a) Monthly average temperature (b) Monthly maximal temperature (c) Monthly minimal temperature: Data were from meteorological stations at Melgueil INRA experimental station.**
(TIF)

Table S1 **Mean phenotypic values and standard deviations (SD) for the parents ‘Olivière and ‘Arbequina’ and the value range for the progeny.**
(DOC)

Table S2 **Variance and correlation values between successive years of growth for the total yield per tree.**
(DOC)

Table S3 **Correlations between reproductive traits on the basis of mean phenotypic values per genotype and year scale: (a) Correlations between flowering and fruiting traits at GUs (b) Correlations between Fruit weight per tree and flowering and fruiting traits at GUs.**
(DOC)

Table S4 **Parameters associated with the QTLs detected separately by multiple QTL mapping (MQM) on ‘Olivière’ and ‘Arbequina’ parental maps, for the best linear unbiased predictors (BLUPs) of reproductive traits measured at both tree and GUs scale over 4 and 3 years, respectively.** For each trait, QTLs detected for the genotype effect BLUP were presented first followed by those detected for the Year-specific BLUPs.
(DOC)

Table S5 **Correlations betweens reproductive growth traits on the bases of G and (GxY) BLUPs.**
(DOC)

Text S1 **QTL detection and mapping on parental maps.**
(DOC)
