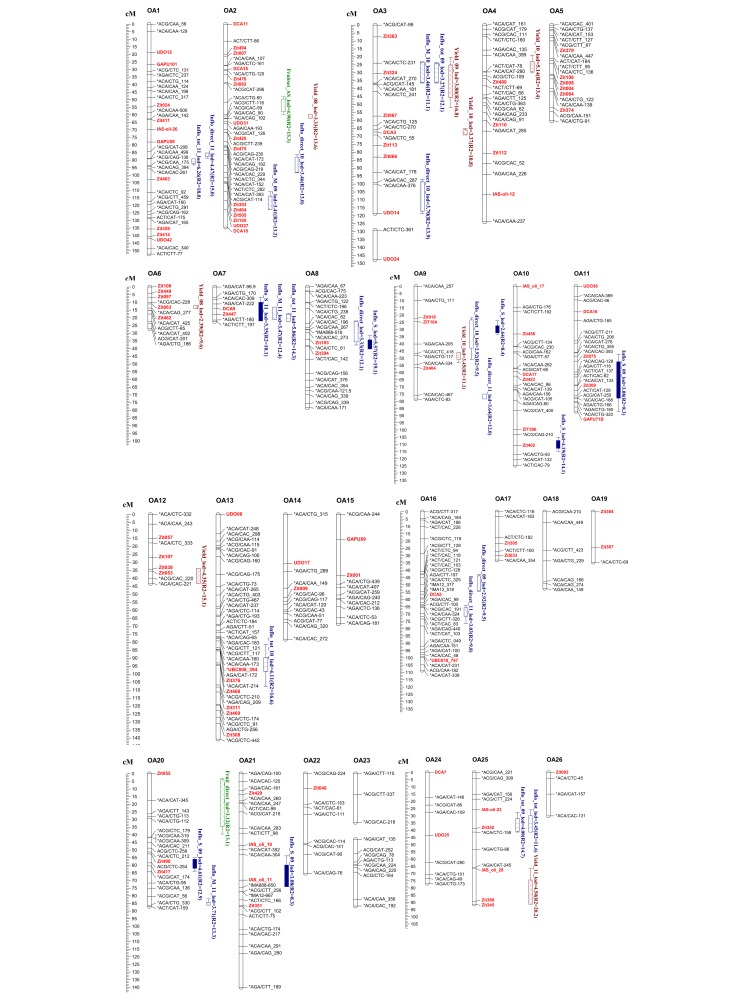# Correction: QTL Mapping of Flowering and Fruiting Traits in Olive

**DOI:** 10.1371/annotation/84425885-b6dc-40b9-b639-422c231cc97e

**Published:** 2014-01-06

**Authors:** Inès Ben Sadok, Jean-Marc Celton, Laila Essalouh, Amal Zine El Aabidine, Gilbert Garcia, Sebastien Martinez, Naziha Grati-Kamoun, Ahmed Rebai, Evelyne Costes, Bouchaib Khadari

Only a portion of Figure 5 is included in the article. Please find a full version of Figure 5 here: 

**Figure:**