# Supplementary figures and images for: QTL Mapping of Flowering and Fruiting Traits in Olive
Source: PLoS One. 2013 May 17;8(5):e62831. doi: 10.1371/journal.pone.0062831 (PMC3656886; doi:10.1371/journal.pone.0062831)

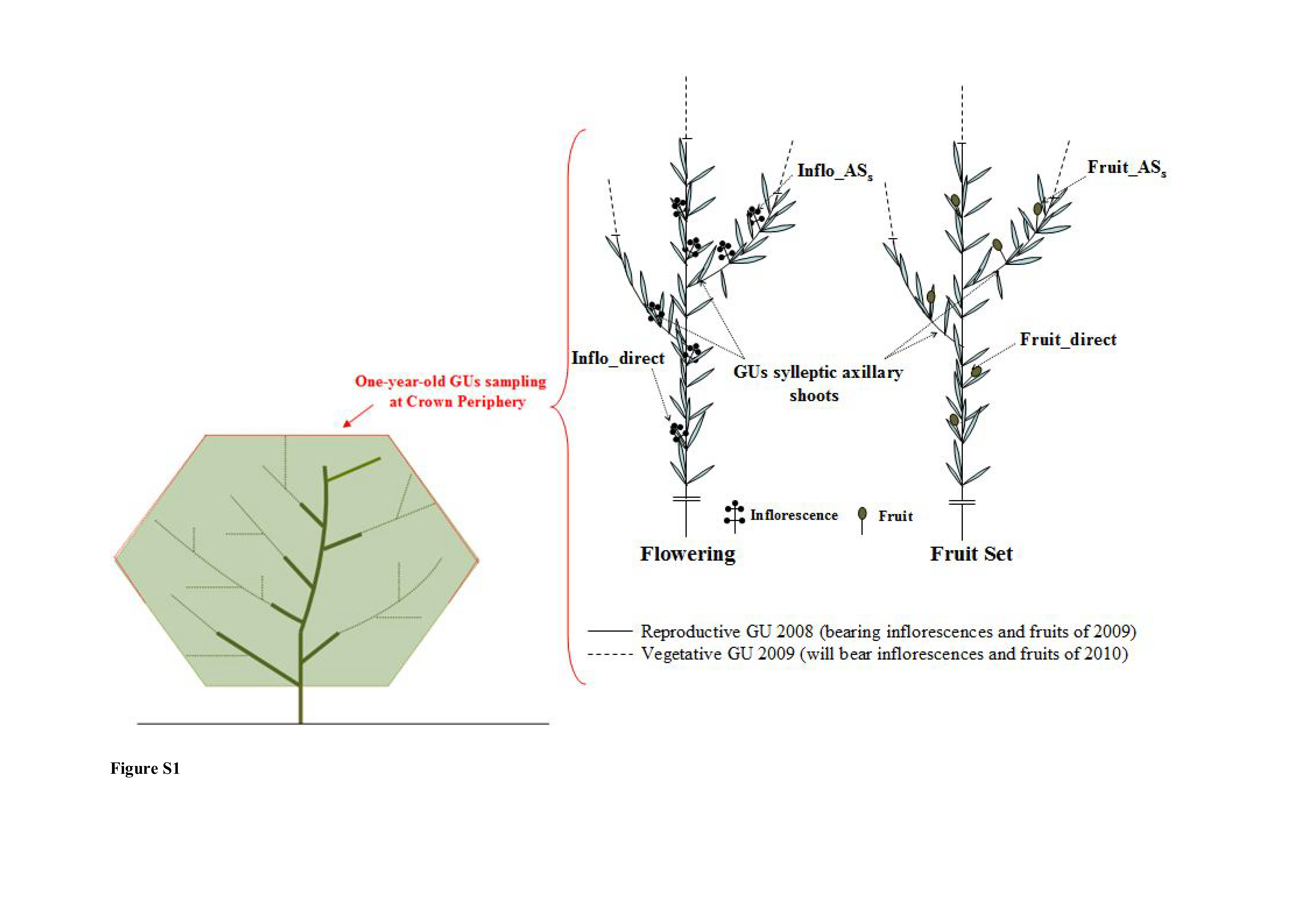

Supplement: Figure S1 — Schematic representation of a 5-year-old olive tree phenotyped in 2009: flowering and fruiting traits collected on 1-year-old GUs at flowering and fruit set periods; the number of inflorescences and fruits born along the floral GUs (Inflo(Fruit)_direct) or along their sylleptic laterals (Inflo(Fruit)_AS) were counted. Floral buds in year i (2009 in the present case) are induced during the summer of year i-1 (2008 in our case) and were thus born on GUs of year i-1. Their final differentiation occurs in year i [14]. (TIF) [file pone.0062831.s001.tif]

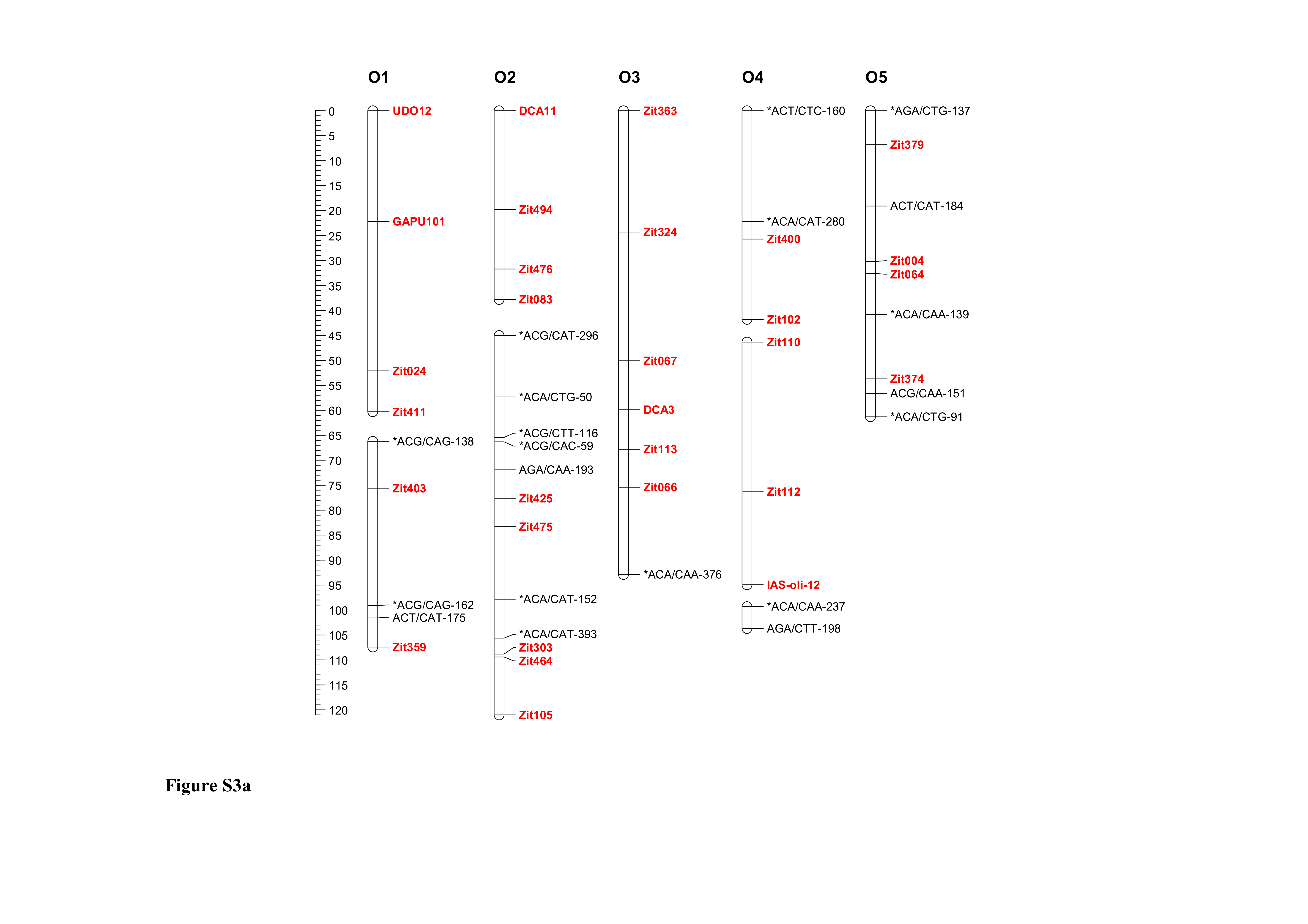

Supplement: Figure S3 — Representation of parental genetic linkage maps (a) ‘Olivière’ female parent map (b) ‘Arbequina’ male parent map. Map distances were derived using the Kosambi mapping function. AFLP markers presented without ‘*’are segregating in both parents. SSRs markers are colored in red (TIF) [file pone.0062831.s003.tif]

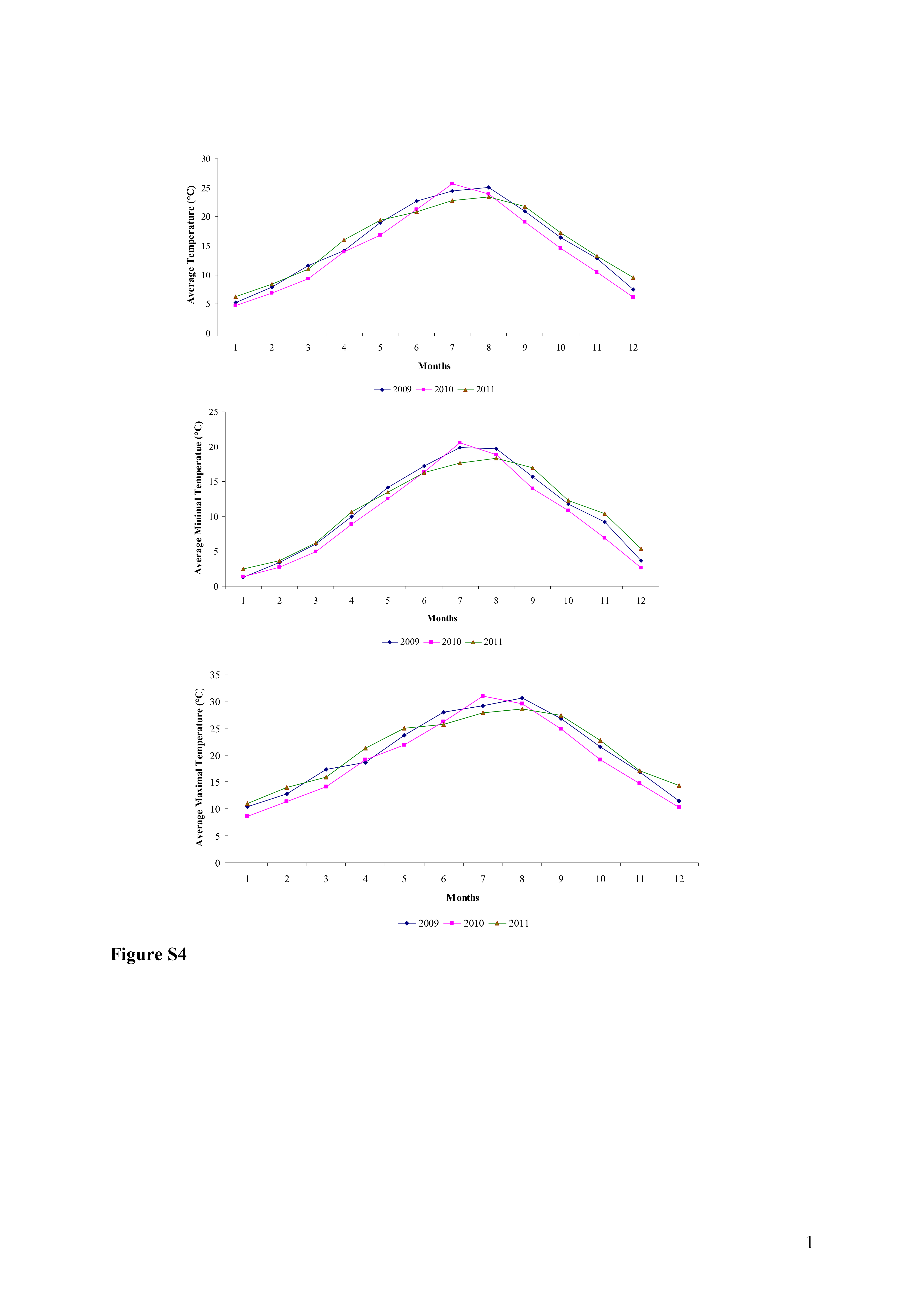

Supplement: Figure S4 — Temperature records during 2009–2011: (a) Monthly average temperature (b) Monthly maximal temperature (c) Monthly minimal temperature: Data were from meteorological stations at Melgueil INRA experimental station. (TIF) [file pone.0062831.s004.tif]
