# Supplementary material for: QTL Mapping of Flowering and Fruiting Traits in Olive
Source: PLoS One. 2013 May 17;8(5):e62831. doi: 10.1371/journal.pone.0062831 (PMC3656886; doi:10.1371/journal.pone.0062831)
Supplement: Table S1 — Mean phenotypic values and standard deviations (SD) for the parents ‘Olivière and ‘Arbequina’ and the value range for the progeny. (DOC) [file pone.0062831.s005.doc]

**Table S1**

| **Trait** | **Mean (SD)** | | **Progeny Range** | |
| --- | --- | --- | --- | --- |
|  | **Olivière** | **Arbequina** | **Minimum** | **Maximum** |
| **Tree Scale** |  |  |  |  |
| Yield (Kg) | 2,08 (1,34) | 4,66 (2,05) | 0 | 24,5 |
| **GU scale** |  |  |  |  |
| *Flowering* |  |  |  |  |
| Inflo_tot | 11,91 (4,75) | 42,08 (36,33) | 1 | 152 |
| Inflo_direct | 7,5 (6,00) | 11,58 (7,80) | 0 | 39 |
| Inflo_AS | 4,42 (6,24) | 30,5 (41,30) | 0 | 143 |
| Inflo_L | 0 | 0 | 0 | 113 |
| Inflo_M | 1,16 (3,73) | 22,16 (32,58) | 0 | 96 |
| Inflo_S | 3,25 (4,71) | 8,33 (10,05) | 0 | 61 |
| *Fruiting* |  |  |  |  |
| Fruit_tot | 4 (4,97) | 18,16 (23,22) | 0 | 123 |
| Fruit_direct | 2,25 (3,74) | 5,58 (4,75) | 0 | 58 |
| Fruit_AS | 1,75 (4,09) | 12,58 (22,94) | 0 | 98 |
| Total_Fruitset (%) | 29,58 (32,89) | 33,58 (19,41) | 0 | 600 |
| Fruitset_direct (%) | 21,66 (31,38) | 102,83 (153,54) | 0 | 700 |
| Fruitset_AS (%) | 12,33 (25,39) | 17 (23,61) | 0 | 300 |
