# Supplementary material for: QTL Mapping of Flowering and Fruiting Traits in Olive
Source: PLoS One. 2013 May 17;8(5):e62831. doi: 10.1371/journal.pone.0062831 (PMC3656886; doi:10.1371/journal.pone.0062831)
Supplement: Table S2 — Variance and correlation values between successive years of growth for the total yield per tree. (DOC) [file pone.0062831.s006.doc]

**Table S2**

| **variances** | **2008** | **2009** | **2010** | **2011** |
| --- | --- | --- | --- | --- |
| **2008** | 793.00998 | 136.79404 | 120.75999 | -11.67654 |
| **2009** | 136.79404 | 972.17294 | 90.67249 | 63.40609 |
| **2010** | 120.75999 | 90.67249 | 775.54128 | 50.87256 |
| **2011** | -11.67654 | 63.40609 | 50.87256 | 1286.29039 |
| **correlations** | **2008** | **2009** | **2010** | **2011** |
| **2008** | - | 0.12524766 | 0.12074616 | -0.01952145 |
| **2009** | 0.12524766 | - | 0.08644844 | 0.03221859 |
| **2010** | 0.12074616 | 0.08644844 | - | 0.03105333 |
| **2011** | -0.01952145 | 0.03221859 | 0.03105333 | - |
