# Supplementary material for: QTL Mapping of Flowering and Fruiting Traits in Olive
Source: PLoS One. 2013 May 17;8(5):e62831. doi: 10.1371/journal.pone.0062831 (PMC3656886; doi:10.1371/journal.pone.0062831)
Supplement: Table S3 — Correlations between reproductive traits on the basis of mean phenotypic values per genotype and year scale: (a) Correlations between flowering and fruiting traits at GUs (b) Correlations between Fruit weight per tree and flowering and fruiting traits at GUs. (DOC) [file pone.0062831.s007.doc]

**Table S3a**

|  | Inflotot09 | Inflodirect09 | InfloAS09 | InfloL09 | InfloM09 | InfloS09 | Fruittot09 | Totalfruitset09 | fruitsetdirect09 | fruitsetAS09 | Inflotot10 | Inflodirect10 | InfloAS10 | InfloL10 | InfloM10 | InfloS10 | Fruittot10 | Totalfruitset10 | fruitsetdirect10 | fruitsetAS10 | Inflotot11 | Inflodirect11 | InfloAS11 | InfloM11 | InfloS11 | Fruittot11 | Totalfruitset11 | fruitsetdirect11 | fruitsetAS11 |
| --- | --- | --- | --- | --- | --- | --- | --- | --- | --- | --- | --- | --- | --- | --- | --- | --- | --- | --- | --- | --- | --- | --- | --- | --- | --- | --- | --- | --- | --- |
| Inflotot09 | - |  |  |  |  |  |  |  |  |  |  |  |  |  |  |  |  |  |  |  |  |  |  |  |  |  |  |  |  |
| Inflodirect09 | ***0,42*** | - |  |  |  |  |  |  |  |  |  |  |  |  |  |  |  |  |  |  |  |  |  |  |  |  |  |  |  |
| InfloAS09 | ***0,96*** | 0,14 | - |  |  |  |  |  |  |  |  |  |  |  |  |  |  |  |  |  |  |  |  |  |  |  |  |  |  |
| InfloL09 | 0,47 | 0,07 | 0,49 | - |  |  |  |  |  |  |  |  |  |  |  |  |  |  |  |  |  |  |  |  |  |  |  |  |  |
| InfloM09 | 0,79 | 0,07 | 0,84 | 0,23 | - |  |  |  |  |  |  |  |  |  |  |  |  |  |  |  |  |  |  |  |  |  |  |  |  |
| InfloS09 | 0,59 | 0,15 | 0,60 | -0,02 | 0,22 | - |  |  |  |  |  |  |  |  |  |  |  |  |  |  |  |  |  |  |  |  |  |  |  |
| Fruittot09 | 0,26 | 0,08 | 0,26 | 0,18 | 0,29 | 0,00 | - |  |  |  |  |  |  |  |  |  |  |  |  |  |  |  |  |  |  |  |  |  |  |
| Totalfruitset09 | **-0,32** | -0,02 | -0,34 | -0,02 | -0,24 | -0,37 | 0,48 | - |  |  |  |  |  |  |  |  |  |  |  |  |  |  |  |  |  |  |  |  |  |
| fruitset direct09 | -0,18 | 0,04 | -0,21 | -0,04 | -0,13 | -0,24 | 0,62 | **0,89** | - |  |  |  |  |  |  |  |  |  |  |  |  |  |  |  |  |  |  |  |  |
| fruitsetAS09 | -0,10 | -0,21 | -0,04 | 0,07 | 0,00 | -0,13 | 0,77 | 0,47 | 0,48 | - |  |  |  |  |  |  |  |  |  |  |  |  |  |  |  |  |  |  |  |
| Inflotot10 | 0,06 | 0,13 | 0,03 | -0,11 | -0,07 | 0,25 | -0,35 | -0,20 | -0,24 | -0,33 | - |  |  |  |  |  |  |  |  |  |  |  |  |  |  |  |  |  |  |
| Inflodirect10 | 0,06 | 0,41 | -0,07 | -0,01 | -0,15 | 0,08 | -0,14 | -0,04 | -0,02 | -0,16 | ***0,54*** | - |  |  |  |  |  |  |  |  |  |  |  |  |  |  |  |  |  |
| InfloAS10 | 0,04 | -0,10 | 0,07 | -0,13 | 0,01 | 0,24 | -0,33 | -0,22 | -0,26 | -0,29 | ***0,85*** | 0,02 | - |  |  |  |  |  |  |  |  |  |  |  |  |  |  |  |  |
| InfloL10 | 0,06 | -0,02 | 0,07 | -0,05 | 0,06 | 0,10 | -0,19 | -0,12 | -0,14 | -0,16 | 0,34 | -0,04 | 0,42 | - |  |  |  |  |  |  |  |  |  |  |  |  |  |  |  |
| InfloM10 | 0,06 | -0,04 | 0,08 | -0,07 | 0,06 | 0,14 | -0,27 | -0,19 | -0,23 | -0,23 | 0,66 | -0,08 | 0,83 | 0,40 | - |  |  |  |  |  |  |  |  |  |  |  |  |  |  |
| InfloS10 | 0,00 | -0,13 | 0,04 | -0,14 | -0,04 | 0,25 | -0,27 | -0,17 | -0,21 | -0,25 | 0,76 | 0,11 | 0,83 | 0,17 | 0,40 | - |  |  |  |  |  |  |  |  |  |  |  |  |  |
| Fruittot10 | -0,05 | 0,22 | -0,12 | 0,01 | -0,11 | -0,11 | 0,35 | 0,44 | 0,47 | 0,20 | -0,01 | 0,24 | -0,15 | -0,11 | -0,13 | -0,12 | - |  |  |  |  |  |  |  |  |  |  |  |  |
| Totalfruitset10 | -0,12 | 0,06 | -0,15 | -0,03 | -0,04 | -0,23 | 0,42 | 0,54 | 0,52 | 0,28 | **-0,38** | -0,19 | -0,34 | -0,16 | -0,27 | -0,28 | 0,70 | - |  |  |  |  |  |  |  |  |  |  |  |
| fruitset direct10 | -0,13 | 0,03 | -0,15 | -0,04 | -0,05 | -0,22 | 0,42 | 0,53 | 0,51 | 0,28 | -0,36 | -0,23 | -0,29 | -0,16 | -0,22 | -0,25 | 0,71 | **0,98** | - |  |  |  |  |  |  |  |  |  |  |
| fruitsetAS10 | -0,14 | -0,08 | -0,13 | 0,00 | -0,09 | -0,15 | -0,06 | 0,01 | -0,08 | -0,02 | 0,17 | -0,12 | 0,28 | 0,03 | 0,28 | 0,21 | 0,25 | 0,10 | 0,17 | - |  |  |  |  |  |  |  |  |  |
| Inflotot11 | 0,00 | 0,27 | -0,08 | -0,04 | -0,09 | -0,01 | -0,07 | 0,02 | 0,00 | -0,19 | 0,43 | 0,42 | 0,25 | 0,07 | 0,19 | 0,23 | 0,14 | -0,12 | -0,09 | 0,07 | - |  |  |  |  |  |  |  |  |
| Inflodirect11 | 0,19 | 0,45 | 0,06 | 0,00 | 0,01 | 0,12 | -0,10 | -0,06 | -0,10 | -0,18 | 0,40 | 0,42 | 0,22 | 0,15 | 0,29 | 0,07 | 0,04 | -0,22 | -0,21 | -0,04 | ***0,74*** | - |  |  |  |  |  |  |  |
| InfloAS11 | -0,19 | -0,06 | -0,19 | -0,06 | -0,15 | -0,14 | 0,00 | 0,10 | 0,10 | -0,10 | 0,23 | 0,20 | 0,15 | -0,06 | -0,02 | 0,28 | 0,18 | 0,05 | 0,08 | 0,14 | ***0,71*** | 0,06 | - |  |  |  |  |  |  |
| InfloM11 | -0,11 | 0,03 | -0,13 | -0,11 | -0,07 | -0,10 | 0,01 | 0,13 | 0,08 | -0,11 | 0,14 | 0,09 | 0,11 | -0,02 | 0,01 | 0,17 | 0,23 | 0,22 | 0,25 | 0,14 | 0,60 | 0,06 | 0,83 | - |  |  |  |  |  |
| InfloS11 | -0,21 | -0,13 | -0,19 | 0,00 | -0,19 | -0,13 | -0,01 | 0,03 | 0,08 | -0,06 | 0,24 | 0,23 | 0,14 | -0,07 | -0,05 | 0,29 | 0,07 | -0,12 | -0,10 | 0,11 | 0,61 | 0,04 | 0,86 | 0,43 | - |  |  |  |  |
| Fruittot11 | -0,07 | 0,14 | -0,12 | -0,01 | -0,11 | -0,09 | 0,42 | 0,43 | 0,45 | 0,30 | -0,11 | 0,01 | -0,14 | -0,16 | -0,17 | -0,04 | 0,63 | 0,53 | 0,57 | 0,18 | 0,27 | 0,07 | 0,33 | 0,39 | 0,18 | - |  |  |  |
| Totalfruitset11 | -0,15 | -0,05 | -0,14 | 0,05 | -0,11 | -0,19 | 0,43 | 0,44 | 0,48 | 0,42 | -0,33 | -0,23 | -0,25 | -0,12 | -0,22 | -0,19 | 0,47 | 0,59 | 0,60 | 0,16 | **-0,31** | -0,39 | -0,05 | 0,02 | -0,11 | 0,73 | - |  |  |
| fruitset direct11 | -0,16 | -0,06 | -0,16 | 0,03 | -0,12 | -0,20 | 0,44 | 0,47 | 0,50 | 0,40 | -0,33 | -0,24 | -0,24 | -0,13 | -0,22 | -0,18 | 0,47 | 0,63 | 0,64 | 0,18 | -0,25 | -0,39 | 0,04 | 0,12 | -0,05 | 0,77 | **0,98** | - |  |
| fruitsetAS11 | -0,11 | -0,04 | -0,11 | -0,10 | -0,17 | 0,08 | 0,17 | 0,24 | 0,36 | 0,14 | 0,15 | 0,00 | 0,18 | -0,07 | 0,05 | 0,28 | 0,24 | 0,17 | 0,17 | 0,14 | 0,07 | -0,17 | 0,29 | 0,22 | 0,26 | 0,46 | 0,35 | 0,37 | - |

**Table S3b**

|  | Yield08 | Yield09 | Yield10 | Yield11 |
| --- | --- | --- | --- | --- |
| Yield08 | - |  |  |  |
| Yield09 | 0,25 | - |  |  |
| Yield10 | 0,51 | 0,27 | - |  |
| Yield11 | 0,04 | 0,49 | 0,08 | - |
| Inflotot09 | -0,07 | 0,00 | -0,20 | -0,02 |
| Inflodirect09 | -0,01 | 0,02 | -0,09 | -0,05 |
| InfloAS09 | -0,07 | 0,00 | -0,18 | 0,00 |
| InfloL09 | 0,08 | 0,09 | -0,04 | 0,17 |
| InfloM09 | **-0,17** | -0,10 | -0,18 | -0,14 |
| InfloS09 | 0,02 | 0,06 | -0,11 | 0,07 |
| Fruittot09 | 0,09 | **0,19** | 0,04 | 0,13 |
| Totalfruitset09 | -0,01 | 0,04 | 0,01 | 0,02 |
| fruitset direct09 | 0,05 | 0,11 | 0,09 | 0,02 |
| fruitsetAS09 | 0,08 | **0,21** | 0,00 | 0,19 |
| Inflotot10 | 0,11 | **-0,15** | -0,06 | -0,04 |
| Inflodirect10 | 0,27 | 0,06 | 0,01 | 0,11 |
| InfloAS10 | -0,03 | **-0,21** | -0,07 | -0,11 |
| InfloL10 | -0,04 | -0,10 | -0,02 | -0,11 |
| InfloM10 | 0,00 | -0,10 | -0,02 | -0,18 |
| InfloS10 | -0,05 | **-0,24** | -0,10 | 0,00 |
| Fruittot10 | 0,15 | **0,34** | **0,15** | 0,01 |
| Totalfruitset10 | -0,01 | **0,26** | 0,07 | -0,04 |
| fruitset direct10 | -0,02 | **0,26** | 0,06 | -0,03 |
| fruitsetAS10 | 0,09 | 0,07 | 0,10 | 0,04 |
| Inflotot11 | -0,01 | -0,09 | **-0,17** | 0,10 |
| Inflodirect11 | -0,01 | -0,05 | **-0,18** | **0,13** |
| InfloAS11 | -0,01 | -0,08 | -0,06 | 0,01 |
| InfloM11 | -0,09 | -0,07 | -0,13 | 0,02 |
| InfloS11 | 0,07 | -0,07 | 0,02 | -0,01 |
| Fruittot11 | 0,07 | 0,13 | 0,10 | **0,12** |
| Totalfruitset11 | 0,13 | 0,12 | **0,23** | 0,01 |
| fruitset direct11 | 0,11 | 0,11 | **0,22** | 0,00 |
| fruitsetAS11 | 0,07 | 0,03 | 0,14 | 0,04 |
