# Supplementary material for: QTL Mapping of Flowering and Fruiting Traits in Olive
Source: PLoS One. 2013 May 17;8(5):e62831. doi: 10.1371/journal.pone.0062831 (PMC3656886; doi:10.1371/journal.pone.0062831)
Supplement: Table S4 — Parameters associated with the QTLs detected separately by multiple QTL mapping (MQM) on ‘Olivière’ and ‘Arbequina’ parental maps, for the best linear unbiased predictors (BLUPs) of reproductive traits measured at both tree and GUs scale over 4 and 3 years, respectively. For each trait, QTLs detected for the genotype effect BLUP were presented first followed by those detected for the Year-specific BLUPs. (DOC) [file pone.0062831.s008.doc]

**Table S4**

| **BLUP** | **Linkage Group** | **LOD^a^** | **Var (%)^b^** | **Female**  **Additivity^c^** | **Male**  **Additivity^c^** | **Cofactor^d^** | **Global R²^e^**  (p-value) |
| --- | --- | --- | --- | --- | --- | --- | --- |
| **Tree Scale** |  |  |  |  |  |  |  |
| Yield | O12 | 3.60 (2,5) | 13.7 | 4.5758 |  | *Zit053* | 0.164 |
|  | O3 | 1.95 (1,3) | 8.1 | -3.5208 |  | *ACA/CAA-376 | (0.0005) |
|  | A12 | 2.46 (1,6) | 11.4 |  | -4.1351 | *Zit053* |  |
|  |  |  |  |  |  |  |  |
| Yield_08 | O2.1 | 2.16 (1,8) | 9.3 | -2.8402 |  | *ACG/CAC-59 | 0.164 |
|  | O6 | 1.84 (1,5) | 7.9 | -2.6193 |  | Zit482 | (0.0002) |
|  | A13 | 2.04 (1,5) | 8.8 |  | -2.7545 | *ACG/CTT_117 | 0.149 |
|  | A21 | 1.98 (2.0) | 8.5 |  | 2.713 | *ACA/CAA_283 | (0.0002) |
|  |  |  |  |  |  |  |  |
| Yield_09 | O19 | 2.85 (1,9) | 13.0 | -3.2997 |  | *ACA/CTC-69 |  |
|  | A23 | 2.70 (1,5) | 12.4 |  | -3.2399 | *AGA/CAG_220 |  |
|  |  |  |  |  |  |  |  |
| Yield_10 | O3 | 1.74 (1,7) | 8.2 | -2.5777 |  | *DCA3* |  |
|  | A3 | 2.61 (2,1) | 10.1 |  | 2.9454 | *DCA3* |  |
|  |  |  |  |  |  |  |  |
| Yield_11 | O25 | 3.12 (1,3) | 14.5 | -5.1584 |  | Zit388 |  |
| **GU Scale** |  |  |  |  |  |  |  |
| Inflo_tot | A25 | 2.21 (1,8) | 8,3 |  | 0.03732 | Zit342 | 0.164 |
|  | A13.1 | 2.31 (1,4) | 8,6 |  | 0.03743 | *Zit376* | (0.0008) |
|  | O10 | 2.62 (1,7) | 11,3 | 0.0428 |  | Zit402 |  |
|  |  |  |  |  |  |  |  |
| Inflotot_09 | O3 | 2.13 (1,6) | 9,3 | -0.0987 |  | *Zit324* |  |
|  | A3 | 2.13 (2,0) | 9,3 |  | -0.0988 | *Zit324* |  |
|  |  |  |  |  |  |  |  |
| Inflotot_10 | O13 | **3.80** (1,8) | 15,9 | 0.1184 |  | *Zit376* |  |
|  | A13.1 | **4.17** (1,3) | 16,8 |  | 0.1226 | *Zit376* |  |
|  |  |  |  |  |  |  |  |
| Inflotot_11 | A1 | 2,93 (2,4) | 11,6 |  | 0,0631 | *AGA/CAA_142 | 0.190 |
|  | A7 | 2,52 (1,5) | 9,9 |  | -0,0585 | *ACT/CTT_197 | (3.266e-05) |
|  | O15 | 2.46 (1,5) | 10,6 | 0.0602 |  | *ACG/CAT-259 |  |
|  |  |  |  |  |  |  |  |
| Inflo_direct | A8 | 2.15 (1,9) | 9.3 |  | 0.1128 | *ACT/CAC_142 |  |
|  |  |  |  |  |  |  |  |
| Inflodirect_09 | O16 | 2.53 (1,5) | 10.9 | 0.1385 |  | *ACG/CTC-128 |  |
|  |  |  |  |  |  |  |  |
| Inflodirect_10 | A8 | 2.44 (2) | 10.5 |  | 0.0956 | *ACT/CAC_142 |  |
|  | O9.1 | 1.54 (1,3) | 6.2 | -0.0731 |  | *AGA/CTG-117 |  |
|  |  |  |  |  |  |  |  |
| Inflodirect_11 | O9.1 | 2.53 (1,4) | 9.1 | 0.0676 |  | *AGA/CTC-83 | 0.230 |
|  | O16 | 3.14 (1,4) | 11.5 | -0.0748 |  | *ACG/CTT-326 | (2.894e-06) |
|  | O23 | 2.02 (1,6) | 7.2 | -0.0600 |  | *ACG/CAC-218 |  |
|  |  |  |  |  |  |  |  |
| Inflo_M | O2.1 | 3.14 (1,8) | 10.6 | 0.0360 |  | Zit105 | 0.273 |
|  | O15 | 1.76 (1,5) | 5.8 | -0.0263 |  | *ACA/CTC-53 | (2.531e-07) |
|  | O16 | 1.74 (1,5) | 5.7 | -0.0257 |  | *ACG/CTT-326 |  |
|  | A2 | 2.13 (2,0) | 8.8 |  | -0.0321 | *ACA/CAC_229 |  |

**Table S4** (continued)

| **Trait** | **Linkage Group** | **LOD^a^** | **Var (%)^b^** | **Female**  **Additivity** | **Male**  **Additivity** | **Cofactor^c^** | **Global R²^e^**  (p-value) |
| --- | --- | --- | --- | --- | --- | --- | --- |
| **GU Scale** |  |  |  |  |  |  |  |
| Inflo_M_09 | O2.1 | 2.10 (1,7) | 8.4 | 0.2021 |  | Zit105 | 0.168 |
|  | O15 | 1.94 (1,6) | 7.7 | -0.1886 |  | *ACA/CTC-53 | (5.309e-05) |
|  |  |  |  |  |  |  |  |
| Inflo_M_10 | O3 | 2.07 (1,6) | 9.0 | 0.0980 |  | *Zit324* |  |
|  | A3 | 2.07 (1,9) | 9.0 |  | 0.0980 | *Zit324* |  |
|  |  |  |  |  |  |  |  |
| Inflo_M_11 | O6 | 2.13 (1,3) | 9.3 | -0.0710 |  | Zit063 |  |
|  | A20 | 1.99 (1,9) | 7.3 |  | 0.0638 | *ACA/CTG_330 |  |
|  |  |  |  |  |  |  |  |
| Inflo_S | O10 | 3.00 (1,7) | 12.3 | 0.0179 |  | Zit402 |  |
|  |  |  |  |  |  |  |  |
| Inflo_S_09 | O3 | 1.66 (1,6) | 6.7 | -0.1613 |  | *Zit324* |  |
|  | A21 | 3.04 (2,1) | 12.9 |  | -0.2287 | IAS_oli_11 |  |
|  |  |  |  |  |  |  |  |
| Inflo_S_10 | O15 | 2.44 (1,5) | 8.6 | -0.1335 |  | *ACG/CAT-259 | 0.245 |
|  | O11 | 2.25 (1,8) | 7.9 | 0.1303 |  | *ACA/CAT-276 | (4.466e-05) |
|  | O13 | 1.96 (1,7) | 6.8 | 0.1231 |  | *Zit376* |  |
|  |  |  |  |  |  |  |  |
| Inflo_S_11 | O9.1 | 1.86 (1,4) | 7.7 | -0.0945 |  | *ACA/CAC-467 |  |
|  | A7 | 2.02 (1,5) | 8.3 |  | 0.1010 | Zit447 |  |
|  |  |  |  |  |  |  |  |
| Fruit_tot | O11 | 1.43 (1,8) | 6.3 | -0.1912 |  | *ACA/CAG-128 |  |
|  | A20 | 2.37 (2,1) | 8.9 |  | 0.2322 | *AGA/CTT_143 | 0.194 |
|  | A2 | 2.15 (1,9) | 8.0 |  | 0.2153 | Zit007 | (0.0001) |
|  | A23 | 1.86 (1,6) | 6.9 |  | 0.1990 | *ACG/CAG_78 |  |
|  |  |  |  |  |  |  |  |
| Fruit_direct | A20 | 2.28 (2,1) | 9.9 |  | 0.2284 | *AGA/CTT_143 |  |
|  |  |  |  |  |  |  |  |
| Total_Fruitset | O13 | 2.94 (1.7) | 11.7 | -0.8425 |  | *Zit376* | 0.14 |
|  | O11 | 2.64 (1.7) | 10.4 | -0.8000 |  | *ACA/CAG-128 | (0.001) |
|  | A23 | 1.73 (1,6) | 7.0 |  | 0.6409 | *ACG/CAG_78 | 0.14 |
|  | A13.1 | 1.91 (1,4) | 7.8 |  | -0.6760 | *Zit376* | (0.001) |
|  |  |  |  |  |  |  |  |
| Fruitset_direct | O11 | 2.41 (1,7) | 9.8 | -0.7191 |  | *ACA/CAG-128 | 0.14 |
|  | O13 | 2.32 (1,8) | 9.4 | -0.7002 |  | *Zit376* | (0.001) |
|  | A13.1 | 1.52 (1,5) | 5.8 |  | -0.5408 | *Zit376* | 0.189 |
|  | A20 | 1.96 (2.0) | 7.5 |  | 0.6325 | *AGA/CTT_143 | (0.0001) |
|  | A23 | 1.51 (1,5) | 5.7 |  | 0.5379 | *ACG/CAG_78 |  |
|  |  |  |  |  |  |  |  |
| Fruitset_AS | O13 | 2.50 (1,8) | 10.0 | -0.1534 |  | *ACG/CAG-160 | 0.156 |
|  | O2.1 | 1.95 (1,8) | 7.7 | -0.1369 |  | *ACA/CTG-50 | (9.074e-05) |
|  | A23 | 1.58 (1,4) | 7.0 |  | 0.1280 | *ACG/CAA_224 |  |

^a^ Maximum LOD score value with the considered threshold in parentheses: Bold LOD score values are significant at genome wide threshold.

^b^ Percentage of phenotypic variation explained by the QTL.

^c^markers used as cofactors in the MQM analysis: Italic cofactors are mapped on both parental maps.

^e^ Percentage of variation explained by the global model.
