# Supplementary material for: QTL Mapping of Flowering and Fruiting Traits in Olive
Source: PLoS One. 2013 May 17;8(5):e62831. doi: 10.1371/journal.pone.0062831 (PMC3656886; doi:10.1371/journal.pone.0062831)
Supplement: Table S5 — Correlations betweens reproductive growth traits on the bases of G and (GxY) BLUPs. (DOC) [file pone.0062831.s009.doc]

|  | **Total** | **Fruitset** | **Fruitset** | **Fruit** | **Fruit** | **Inflo** | **Inflo** | **Inflo** | **Inflo** | **Inflo** | **Inflo** | **Inflo** | **Inflo** | **InfloM** | **InfloM** | **InfloM** | **InfloM** | **InfloS** | **InfloS** | **InfloS** | **InfloS** | **Yield** | **Yield** | **Yield** | **Yield** | **Yield** |
| --- | --- | --- | --- | --- | --- | --- | --- | --- | --- | --- | --- | --- | --- | --- | --- | --- | --- | --- | --- | --- | --- | --- | --- | --- | --- | --- |
|  | **Fruitset** | **direct** | **AS** | **tot** | **direct** | **tot** | **tot09** | **tot10** | **tot11** | **direct** | **direct09** | **direct10** | **direct11** |  | **9** | **10** | **11** |  | **9** | **10** | **11** |  | **8** | **9** | **10** | **11** |
| Total_Fruitset | - |  |  |  |  |  |  |  |  |  |  |  |  |  |  |  |  |  |  |  |  |  |  |  |  |  |
| Fruitset_direct | 0,96 | - |  |  |  |  |  |  |  |  |  |  |  |  |  |  |  |  |  |  |  |  |  |  |  |  |
| Fruitset_AS | 0,51 | 0,45 | - |  |  |  |  |  |  |  |  |  |  |  |  |  |  |  |  |  |  |  |  |  |  |  |
| Fruit_tot | 0,92 | 0,91 | 0,55 | - |  |  |  |  |  |  |  |  |  |  |  |  |  |  |  |  |  |  |  |  |  |  |
| Fruit_direct | 0,87 | 0,93 | 0,33 | 0,91 | - |  |  |  |  |  |  |  |  |  |  |  |  |  |  |  |  |  |  |  |  |  |
| Inflo_tot | -0,42 | -0,32 | -0,07 | -0,12 | -0,09 | - |  |  |  |  |  |  |  |  |  |  |  |  |  |  |  |  |  |  |  |  |
| Inflo_tot_09 | -0,13 | -0,08 | -0,10 | 0,02 | 0,01 | **0,54** | - |  |  |  |  |  |  |  |  |  |  |  |  |  |  |  |  |  |  |  |
| Inflo_tot_10 | -0,40 | -0,36 | 0,00 | -0,23 | -0,22 | 0,55 | -0,24 | - |  |  |  |  |  |  |  |  |  |  |  |  |  |  |  |  |  |  |
| Inflo_tot_11 | -0,01 | 0,03 | 0,02 | 0,08 | 0,16 | 0,26 | -0,23 | -0,03 | - |  |  |  |  |  |  |  |  |  |  |  |  |  |  |  |  |  |
| Inflo_direct | -0,09 | 0,05 | -0,26 | 0,12 | 0,35 | 0,66 | 0,28 | 0,32 | 0,38 | - |  |  |  |  |  |  |  |  |  |  |  |  |  |  |  |  |
| Inflo_direct_09 | 0,19 | 0,31 | -0,06 | 0,26 | 0,46 | 0,25 | 0,41 | -0,14 | 0,03 | 0,67 | - |  |  |  |  |  |  |  |  |  |  |  |  |  |  |  |
| Inflo_direct_10 | -0,23 | -0,18 | -0,24 | -0,13 | -0,06 | 0,34 | -0,13 | 0,61 | -0,04 | 0,34 | -0,27 | - |  |  |  |  |  |  |  |  |  |  |  |  |  |  |
| Inflo_direct_11 | -0,21 | -0,27 | -0,01 | -0,12 | -0,20 | 0,24 | -0,12 | 0,03 | **0,66** | 0,01 | **-0,37** | **-0,22** | - |  |  |  |  |  |  |  |  |  |  |  |  |  |
| Inflo_M | -0,32 | -0,28 | 0,06 | -0,19 | -0,28 | 0,52 | 0,57 | 0,05 | 0,02 | 0,01 | 0,04 | -0,17 | 0,16 | - |  |  |  |  |  |  |  |  |  |  |  |  |
| Inflo_M_09 | -0,24 | -0,19 | -0,09 | -0,12 | -0,18 | 0,45 | 0,75 | -0,13 | -0,17 | 0,05 | 0,10 | -0,08 | 0,01 | 0,86 | - |  |  |  |  |  |  |  |  |  |  |  |
| Inflo_M_10 | -0,31 | -0,35 | 0,17 | -0,27 | -0,36 | 0,20 | **-0,19** | 0,43 | 0,05 | -0,11 | -0,16 | -0,20 | 0,37 | 0,29 | -0,12 | - |  |  |  |  |  |  |  |  |  |  |
| Inflo_M_11 | 0,18 | 0,20 | 0,21 | 0,17 | **0,20** | -0,02 | -0,22 | -0,10 | **0,50** | 0,07 | 0,07 | 0,03 | -0,06 | 0,08 | -0,17 | -0,20 | - |  |  |  |  |  |  |  |  |  |
| Inflo_S | -0,36 | -0,34 | 0,32 | -0,18 | -0,33 | 0,54 | 0,37 | 0,31 | -0,01 | -0,06 | -0,10 | -0,04 | **0,15** | 0,37 | 0,25 | 0,29 | -0,03 | - |  |  |  |  |  |  |  |  |
| Inflo_S_09 | -0,27 | -0,25 | 0,00 | -0,10 | **-0,19** | 0,49 | 0,67 | 0,01 | -0,16 | 0,10 | 0,05 | -0,01 | 0,09 | 0,28 | 0,35 | 0,05 | **-0,25** | 0,71 | - |  |  |  |  |  |  |  |
| Inflo_S_10 | -0,26 | -0,30 | 0,34 | -0,20 | -0,33 | 0,22 | -0,13 | 0,52 | -0,15 | -0,22 | -0,17 | -0,15 | 0,12 | 0,19 | -0,02 | 0,52 | -0,10 | 0,62 | 0,06 | - |  |  |  |  |  |  |
| Inflo_S_11 | -0,05 | 0,00 | 0,32 | 0,00 | -0,04 | 0,16 | -0,14 | 0,07 | **0,47** | -0,03 | -0,13 | 0,12 | 0,05 | 0,16 | 0,02 | -0,07 | **0,52** | 0,35 | -0,14 | 0,09 | - |  |  |  |  |  |
| Yield | 0,64 | 0,65 | 0,33 | 0,69 | 0,69 | 0,03 | 0,01 | -0,07 | 0,18 | 0,31 | 0,29 | 0,06 | -0,12 | -0,14 | -0,06 | -0,34 | 0,18 | -0,30 | -0,22 | -0,30 | 0,02 | - |  |  |  |  |
| Yield_08 | 0,02 | 0,06 | -0,02 | 0,05 | 0,15 | 0,32 | 0,10 | 0,24 | 0,14 | 0,40 | 0,15 | 0,44 | -0,16 | 0,08 | 0,17 | -0,26 | 0,09 | -0,09 | -0,27 | -0,01 | 0,28 | 0,21 | - |  |  |  |
| Yield_09 | 0,11 | 0,07 | 0,05 | 0,14 | 0,09 | 0,13 | **0,28** | -0,14 | 0,02 | 0,06 | 0,13 | -0,21 | 0,11 | 0,15 | 0,20 | -0,01 | -0,12 | 0,02 | 0,23 | -0,13 | -0,20 | 0,36 | -0,22 | - |  |  |
| Yield_10 | 0,05 | 0,06 | -0,13 | 0,06 | 0,05 | -0,03 | -0,05 | 0,19 | -0,32 | 0,06 | 0,06 | **0,31** | -0,41 | -0,17 | -0,11 | -0,14 | -0,03 | -0,11 | -0,08 | -0,12 | 0,02 | 0,17 | 0,15 | -0,28 | - |  |
| Yield_11 | 0,39 | 0,38 | 0,35 | 0,37 | 0,34 | **-0,28** | **-0,22** | -0,27 | 0,24 | -0,14 | 0,00 | -0,37 | 0,23 | -0,14 | -0,23 | 0,03 | 0,17 | -0,11 | -0,06 | -0,06 | -0,08 | 0,43 | -0,45 | -0,01 | -0,43 | - |

**Table S5**
