# Supplementary material for: QTL Mapping of Flowering and Fruiting Traits in Olive
Source: PLoS One. 2013 May 17;8(5):e62831. doi: 10.1371/journal.pone.0062831 (PMC3656886; doi:10.1371/journal.pone.0062831)
Supplement: Text S1 — QTL detection and mapping on parental maps. (DOC) [file pone.0062831.s010.doc]

**QTL detection and mapping on parental maps**

QTL detected on the integrated map as well as parental maps are indicated in bold in Table 6. Here are detailed the QTL not found on the integrated map but, detected on the parental maps (Table S4).

**Reproductive traits measured at tree scale**

*Yield*

Besides the QTL detected on O12 (i.e. also found in the integrated map on OA12), a QTL was detected on O3 for the total fruit weight per tree (Yield, Table S4), which explained 8.8 % of the variability. A global model was built for these QTL showing non significant interaction between cofactors and that explained 16.4% of the variability (Table S4).

Two 2008 specific QTLs were detected on A13 and A21 (Yield_08, Table S4) which explained 8.8 % and 8.5% of the variability, respectively. Together, these QTL explained 14.9% of the variability (Table S4). Two 2009 specific QTLs were detected on O19 and A23 (Yield_09, Table S4), which explained 13% and 12.4% of the variability, respectively.

**Reproductive traits measured at GU scale**

*The total number of inflorescences*

In addition to the QTL detected on A25 (i.e. found on OA25 in the integrated map), two QTLs were identified on A13.1 and O10 for the total number of inflorescences per GU (Inflo_tot, Table S4), which explained 8.6% and 11.3% of the variability, respectively. The global model did not include any interaction between the QTL on A25 and A13.1 and explained 16.4% of the variability (Table S4). An additional 2011 specific QTL was detected on O15 and explained 10.6% of the variability (Inflotot_11, Table S4).

*The number of inflorescences* *born at the leaf axils along GUs*

A 2010 specific QTL was found on A8 for Inflo_direct (Inflodirect_10, Table S3), which explained 9.3% of the variability. This QTL co-localized with the QTL previously detected for the G effect BLUP of Inflo_direct (Table S4). In addition to the two QTL previously found on O9.1 and O16 (i.e. OA9 and OA16 in the integrated map), a 2011 specific QTL was found on O23, which explained 7.2% of the variability (Inflodirect_11, Table S4). Together, these QTL explained 23% of the variability (Table S4).

*The number of inflorescences born along GUs medium and short laterals*

Four QTLs were detected for the number of inflorescences per medium laterals on parental maps only (Inflo_M, Table S4). Two of them were mapped on O2.1 and A2 and explained 10.6% and 8.8% of the variability, respectively. The two others were found on O15 and O16, which explained 5.8 % and 5.7% of the variability, respectively. These QTL co-localized with the QTL previously detected for the 2011 specific BLUP of Inflo_tot and Inflo_direct, respectively (Table S4). A global model was built for the three QTL found on ‘Olivière’ map, which explained 27.3% of the variability (Table S4). Further than the QTL found on O2.1 (i.e. OA2 in the integrated map), a 2009 specific QTL was detected on O15 and explained 7.7% of the variability. This QTL co-localized with the previously detected QTL for the G effect BLUP of Inflo_M (Table S4). The global model did not include any interaction between the QTL and explained 16.8% of the variability (Table S4). Considering the 2011 BLUPs, a QTL was detected on O6 for Inflo_M (Inflo_M_11, Table S4), which explained 9.3% of the variability.

A QTL was detected on O3 for 2009 BLUPs of the number of inflorescences per short laterals (Inflo_S_09, Table S4), which explained 6.7% of the variability. This QTL co-localized with the QTL previously detected for Inflotot_09 and Inflo_M_10 (Table S4). Three 2010 specific QTLs were detected on O15, O11 and O13 (Inflo_S_10, Table S4), which explained 8.6%, 7.9% and 6.8% of the variability, respectively. Together, these QTL explained 24.5% of the variability (Table S4).The QTL mapped on O13 co-localized with the previously detected QTL for the 2010 BLUPs of Inflo_tot (Table S4). A 2011 specific QTL was detected on O9.1, which explained 7.7% of the variability (Inflo_S_11, Table S4). This QTL co-localized with the 2011 specific QTLs previously detected for Inflo_direct (Table S4).

*The total number of fruits*

Four QTLs were detected on A20, A2, A23 and O11 for the total number of fruits per GU (Fruit_tot, Table S4). These QTLs explained 8.9%, 8%, 6.9% and 6.3% of the variability, respectively. A global model was built for the three QTL found on ‘Arbéquina’ map, which did not include any interaction between the QTL and explained 19.4% of the variability (Table S4).

*The total fruit set*

Three QTLs were detected on parental maps for the percentage of total fruit set (Total_Fruitset, Table S4). The first QTLs was detected on both parental maps and mapped on O13 and A13.1, which explained 11.7% and 7.8% of the variability. These QTL co-localized with those previously detected for the 2010 BLUPs of Inflo_tot (Table S4). The two others were detected on O11 and A23 and explained 10.4% and 7% of the variability. These QTL co-localized with those previously detected for Fruit_tot (Table S4). A global model was built for the QTL of each parental map, which did not include any interaction between the QTL. Together, the QTL found on each parental map explained 14% of the variability (Table S4).

*The fruit set at the leaf axils along GUs*

Four QTLs were detected for the percentage of direct fruit set (Fruitset_direct, Table S.4). The first three QTLs were mapped on O11, O13 and A13.1 and A23, which explained 9.8%, 9.4%, 5.8% and 5.7% of the variability, respectively. These QTL co-localized with those previously detected for the Total_Fruitset (Table S4). The forth QTL was found on A20 and explained 7.5% of the variability. This QTL co-localized with the QTL previously detected for the Fruit_direct (Table S4). A global model was built for the QTL found on ‘Arbéquina’ map, which did not include any interaction between the QTL and explained 18.9% of the variability (Table S4).

*The fruit set at GUs syllpetic laterals*

In addition to the QTL detected on O2.1 (i.e. OA2 in the integrated map), two QTL were detected on O13 and A23 for the percentage of axillary fruit set (Fruitset_AS, Table S4), which explained 10% and 7% of the variability, respectively. These QTL co-localized with those previously detected for Total_Fruitset and Fruitset_direct (Table S4). Together, the QTL found on ‘Olivière’ map explained 15.6% of the variability (Table S4).
